# Is There a Difference in the Proteomic Profile of Stimulated and Unstimulated Saliva Samples from Pregnant Women with/without Obesity and Periodontitis?

**DOI:** 10.3390/cells12101389

**Published:** 2023-05-14

**Authors:** Gerson Aparecido Foratori-Junior, Talita Mendes Oliveira Ventura, Larissa Tercilia Grizzo, Bruno Gualtieri Jesuino, Ana Virgínia Santana Sampaio Castilho, Marília Afonso Rabelo Buzalaf, Silvia Helena de Carvalho Sales-Peres

**Affiliations:** 1 Department of Pediatric Dentistry, Orthodontics and Public Health, Bauru School of Dentistry, University of São Paulo, Bauru 17012-901, Brazil; 2 Department of Biological Sciences, Bauru School of Dentistry, University of São Paulo, Bauru 17012-901, Brazil

**Keywords:** obesity, periodontitis, pregnancy, proteomics, saliva

## Abstract

This study aimed to compare the proteomic profile of stimulated and unstimulated saliva samples from pregnant women with/without obesity and periodontitis. Pregnant women were allocated into four groups: with obesity and periodontitis (OP); with obesity but without periodontitis (OWP); with normal BMI but with periodontitis (NP); with normal BMI and without periodontitis (NWP). Stimulated saliva (SS) and unstimulated saliva (US) samples were collected, and salivary proteins were extracted and individually processed by proteomic analysis (nLC-ESI-MS/MS). Proteins involved with the immune response process, antioxidant activity, and retina homeostasis were decreased or absent in SS samples from all groups (i.e., *Antileukoproteinase*, *Lysozyme C*, *Alpha-2-macroglobulin-like protein 1*, *Heat shock proteins—70 kDa 1-like*, *1A*, *1B*, *6*, *Heat shock-related 70 kDa protein 2*, *Putative Heat shock 70 kDa protein 7*, *Heat shock cognate 71 kDa*). Additionally, proteins related to the carbohydrate metabolic process and glycolytic and glucose metabolic process were absent in SS, mainly from OP and OWP (i.e., *Frutose-bisphosphate aldose A*, *Glusoce-6-phosphate isomerase*, *Pyruvate kinase*). Saliva stimulation decreased important proteins involved with immune response and inflammation process in all groups. Unstimulated salivary samples seem to be the best choice for the proteomic approach in pregnant women.

## 1. Introduction

Saliva is a biological fluid composed of more than 99% water and less than 1% protein, electrolytes, and other low molecular weight components [1]. In addition to providing biomarkers for the diagnosis of local and systemic diseases, saliva plays a key role in protecting oral tissues. Saliva collection is a non-invasive and pain-free method; therefore, several studies have sought to better understand the diagnostic potential of saliva through the identification of biomarkers, whether proteins or metabolites. Proteomic approaches based on mass spectrometry have been applied in several fields of biomedical research to identify new biomarkers [2,3,4].

Obesity is one of the major public health problems in low-, middle-, and high-income countries. The 2017 global nutrition report showed that two billion adults are overweight/obese worldwide [5]. Obesity is known to start a broad inflammatory response through the production of cytokines (*interleukins*, IL-1, IL-6, IL-8, and *Tumor Necrosis Factor alpha*, TNF-α), adipokines (*leptin*, *adiponectin*, *resistin*, and inhibitors of plasminogen activator-1), and other bioactive substances, such as Reactive Oxygen Species (ROS), by adipose tissue [6,7,8]. Thus, there is a greater chance of individuals with overweight/obesity to present inflammation in the oral cavity, even in the presence of a small amount of biofilm. Previous studies showed the mechanism by which obesity is linked to an increased inflammatory response to sub and supragingival biofilm, associating obesity with the high prevalence of periodontitis [9].

Evidence shows that both obesity and periodontitis are also associated with low salivary flow rates [10,11]. In the same way, although there is evidence that in the first trimester of pregnancy there is an increase in salivary flow, pregnant women during the second and third trimesters showed lower salivary flow rates when compared to non-pregnant women [12]. In addition, during the third trimester, pregnant women are more prone to periodontal inflammation due to high levels of gestational hormones [13]. In previous studies by our team, we performed proteomics and metabolomics analyses of unstimulated saliva samples from pregnant women with/without obesity and periodontitis during the third trimester of pregnancy [14,15]. One of the challenges was the difficulty in collecting saliva from pregnant women due to frequent nausea and, mainly, the reduced flow in some patients.

Collecting stimulated instead of unstimulated saliva would facilitate clinical practice, both due to the reduced collection time and the ease of obtaining an adequate amount for analysis. However, it is important to assess whether there are no losses related to protein identification in performing proteomic analysis of stimulated saliva in pregnant women with/without obesity and periodontitis. Therefore, this study aimed to compare the proteomic profile of stimulated and unstimulated saliva samples from pregnant women with/without obesity and periodontitis. The null hypothesis was that there were no differences in protein identification in the proteomic analysis of stimulated and unstimulated saliva samples from pregnant women with/without obesity and periodontitis.

## 2. Materials and Methods

This observational, cross-sectional, and analytical study adhered to the STROBE (Strengthening the Reporting of Observational Studies in Epidemiology) standards [16].

### 2.1. Ethical Statement

This study was approved by the Internal Research Ethics Committee of the Bauru School of Dentistry, University of São Paulo (CAAE 06624519.3.0000.5417), being in accordance with the protocol established by the Declaration of Helsinki (published in 1975 and revised in 2013). Following the submission of a formal written consent form, individuals were included.

### 2.2. Sample Selection

Sampling recruitment method, as well as inclusion and exclusion criteria, were described in detail elsewhere [14]. Initially, 126 pregnant women (27th–39th gestational week), 18–40 years old, were selected from the Primary Health Care Units of Bauru, São Paulo, Brazil, between December 2020 and March 2021, but 49 were excluded due to the following reasons: stage I of periodontitis (*n* = 20); overweight (intermediary BMI, 25–30 kg/m^2^, between eutrophics and obesity; *n* = 10); arterial hypertension during pregnancy (*n* = 5); Gestational Diabetes Mellitus (*n* = 4); smoker (*n* = 3); SARS-CoV-2 infection (*n* = 3); multiple tooth loss (*n* = 2); twin pregnancy (*n* = 1); and underweight (BMI < 18.5 kg/m^2^; *n* = 1). These exclusion criteria were adopted to ensure a homogenous sample, avoiding bias in the interpretation of the proteomic analysis. Pregnant women were allocated into four groups according to the presence of obesity and periodontitis: with obesity and periodontitis (OWP = 11), with obesity but without periodontitis (OWP = 27), with normal BMI but with periodontitis (NP = 10), and with normal BMI and without periodontitis (NWP = 29). According to previous in vivo individual salivary proteomic analysis by mass spectrometry [1,14,17], the sample was randomized, and 10 individuals from each group were selected for the final sample (OP = 10; OWP = 10; NP = 10; NWP = 10). As mentioned, in vivo individual salivary proteome mass spectrometry analyses were used to determine the sample size [1,14,17].

### 2.3. Grouping Variables

Grouping method was described elsewhere [14]. Groups OP and OWP were composed of individuals with BMI equal to or higher than 30.00 kg/m^2^, while groups NP and NWP were composed of individuals with BMI between 18.50–24.99 kg/m^2^ [14,15,18,19,20,21,22,23,24].

Periodontal evaluation was performed by one calibrated dentist (kappa = 0.95), who registered the probing pocket depth (PPD) and clinical attachment level (CAL)/attachment loss (AL). Periodontitis classifications and categorizations according to severity described by Tonetti and collaborators (2018) [25] were adopted for sampling. To ensure a more homogenous sample for proteomic analysis, participants classified as having stages I and IV of periodontitis were not considered. To avoid unnecessarily exposing pregnant women to X-rays, only clinical characteristics were used to determine the severity of periodontitis.

### 2.4. Saliva Collection

Saliva collection method was described elsewhere [14]. In summary, in consideration of the circadian rhythm, saliva collection was conducted in the morning (09:00–11:00) and was placed before the periodontal examination. After rinsing their mouths for 1 min with 5 mL of deionized water, the patients were asked to passively drool for 10 min into a sterilized plastic falcon tube (50 mL) immersed in ice (unstimulated whole-mouth saliva—US) [1,14,17]. For the stimulated saliva (SS) collection, patients were instructed to chew a sterilized parafilm gum for 11 min. The saliva produced in the first minute was discarded, and the whole amount produced in the following 10 min was put into a sterilized plastic falcon tube (50 mL) immersed in ice [24]. Saliva volume and flow rate were recorded.

Saliva was centrifuged at 4500× *g* for 15 min at 4 °C immediately following each collection to clear out all debris. Each sample’s supernatant was taken and stored at −80 °C in a freezer until the time of the proteome analysis.

### 2.5. Sample Preparation for Proteomic Analysis

Sample preparation for proteomic analysis was previously described in detail [1,14,17]. In summary, all samples (1000 µL each) were analyzed individually, and the sample preparation was divided into seven steps: (1) Extraction: proteins were extracted using a solution (1000 µL for each sample) containing 6 M urea, 2 M thiourea in 50 mM NH_4_HCO_3_, pH 7.8. Samples were vortexed three times for 10 min at 4 °C, sonicated for 5 min, and centrifuged at 20,817× *g* at 4 °C for 10 min. (2) Concentration: samples were concentrated to a volume of around 150 L in Amicon tubes (Amicon Ultra-15 Centrifugal Filter Units—Merck Millipore^®^, Tullagreen, County Cork, Ireland). (3) Reduction and alkylation: proteins were reduced with 5 mM dithiothreitol for 40 min at 37 °C and alkylated with 10 mM iodoacetamide for 30 min in the dark. (4) Digestion: samples were digested for 14 h at 37 °C by addition of 2% trypsin (*w*/*w*) (Thermo Scientific Pierce Trypsin Protease, Rockford, IL, USA). Digestion was stopped by the addition of 10 μL of 5% Trifluoroacetic acid. (5) Desalination and purification: samples were desalted and purified using C18 Spin columns (Thermo Scientific, Rockford, IL, USA). (6) Quantification: an aliquot of 1 μL was taken from each sample for total protein quantification by the Bradford method (Bio-Rad, Hercules, CA, USA). (7) Resuspension: samples were then resuspended in a solution containing 3% acetonitrile and 0.1% formic acid and subjected to Mass Spectrometry (nanoLC-ESI-MS/MS) (Waters, Manchester, New Hampshire, UK).

### 2.6. Proteomic Analysis by nLC-ESI-MS/MS

Proteomic analysis method was also previously described in detail [1,14,17]. The analysis of peptides was performed in a nanoACQUITY UPLC system (Waters, Manchester, New Hampshire, UK) coupled with a Xevo Q-TOF G2 mass spectrometer (Waters, Manchester, New Hampshire, UK). As aforementioned, for the proteomic analysis of saliva, the samples were analyzed individually. The individual analysis in the mass spectrometer is considered a highly reliable method since the system performs the technical triplicate of each sample. The nanoACQUITY UPLC system is equipped with a Trap Column, responsible for chromatography (Trap Column: 100Å, 5 µm, 180 µm × 200 mm) previously equilibrated with 99.9% phase A (0.1% formic acid in water) at a flow of 5 µL/min and an HSS T3 M-Class type column (analytical column; Acquity UPLC HSS T3 M-Class column 75 μm × 150 mm; 1.8 μm) (Waters, Manchester, New Hampshire, United Kingdom), previously equilibrated with 93% mobile phase A and mobile phase B (0.1% formic acid in ACN). Peptides were separated by a linear gradient of 7–85% mobile phase B for 70 min with 0.35 μL/min flow rate; the column temperature was maintained at 45 °C. In order to collect data using the MSE method at high energy (19–45 V), which enables data capture of both precursor and fragment ions in a single injection, the equipment was operated in positive ionic nanoelectrospray mode. Data acquisition scan range was 50–2000 Da. The lockspray was run with a solution of [Glu1] fibrinopeptide (1 pmol/L) at a flow rate of 0.5 L/min in order to assure precision and reproducibility.

*ProteinLynx GlobalServer* (PLGS) version 3.0.3 software (Waters Co., Manchester, UK) was used to process and search the continuous LC-MSE data. PLGS has a model of data and uses Bayesian Markov Chain Monte Carlo methods to explore the posterior probability density of the model parameters, accumulating the relevant statistics as the method proceeds. Thus, proteins were identified using the software’s ion counting algorithm, and a search was performed on the *Homo sapiens* database (reviewed only, UniProtKB/Swiss-Prot) downloaded in May 2022 by UniProtKB (http://www.uniprot.org/ accessed on 25 May 2022). UniProt was used to analyze each protein by its access number. Repeated and reverse proteins, as well as fragments, were excluded. All proteins identified with a confidence level greater than 95% were included in the quantitative analysis. The difference in expression between stimulated and unstimulated saliva samples for each group was expressed as *p* < 0.05 for the down-regulated proteins and 1-*p* > 0.95 for the up-regulated proteins [1,14,17]. The difference in expression between SS and US for each group was analyzed by *t* test (*p* < 0.05). The relevant comparations were performed (1-SS versus US for OP; 2-SS versus US for OWP; 3-SS versus US for NP; 4-SS versus US for NWP). Proteins were only considered uniquely expressed in the groups when they appeared in at least two of the three replicates of those individuals in each group.

### 2.7. Statistical Analysis and Bioinformatics

Although the sample size has been based on previous studies, the sample size was also calculated with G*Power version 3.1.9.6 (Heinrich-Heine-Universität Düsseldorf, Bavaria, Germany), utilizing information from our prior experiment [14], considering α = 0.05 and 1 − β = 0.8. Effect size was estimated to be 2.18. The estimated number of samples was 4/group. We included 10 volunteers in each group, and all samples were individually analyzed.

In light of the clinical parameters, the Kolmogorov–Smirnov test was used to determine the normality of the variables. Analysis of variance (ANOVA) with Scheffé was then used for quantitative variables with a normal distribution, while Kruskal–Wallis with Dunn was used for quantitative variables without a normal distribution.

In proteomic analysis, the difference in expression between stimulated and unstimulated saliva samples for each group was expressed as *p* < 0.05 for the down-regulated proteins and 1-*p* > 0.95 for the up-regulated proteins [1,14,17]. The difference in expression between SS and US for each group was analyzed by *t* test (*p* < 0.05). The relevant comparations were performed (1-SS versus US for OP; 2-SS versus US for OWP; 3-SS versus US for NP; 4-SS versus US for NWP).

ClueGo^®^ plugins of the Cytoscape^®^ 3.9.1 Software (Institute of Systems Biology, Seattle, WA, USA) were used to analyze the protein categories based on gene ontology (GO) annotation of the broad biological process, molecular function, immune system, and cell component. The functional distribution of proteins identified with differential expression (up- and down-regulated) in the comparison between SS and US for each group was carried out. Terms of significance (κ = 0.04) and distribution were according to the percentage of the number of associated genes. The mass spectrometric proteomic data have been deposited to the ProteomeXchange Consortium via the PRIDE partner repository with the data set identifier PXD040373.

For the protein interaction networks analysis, STRING^®^ database (https://string-db.org/cgi/network.pl accessed on 15 February 2022) was accessed. To understand the biological processes involved only in the analysis of unstimulated saliva, unique proteins found in US samples of each group were analyzed in the STRING^®^ database, establishing the interaction between those unique proteins present in unstimulated saliva of each group during pregnancy.

## 3. Results

The mean age (±SD) of the sample was 27.63 ± 4.57 years old (OP = 32.10 ± 5.02; OWP = 25.00 ± 5.09; NP = 26.00 ± 4.18; NWP = 28.40 ± 4.00). The pre-pregnancy BMI mean (±SD) was 33.6 (±3.16), 34.7 (±3.80), 21.3 (±2.59), and 23.3 (±1.71) kg/m^2^ for OP, OWP, NP, and NWP, respectively, while the pregnancy BMI mean during the third trimester was 36.0 (±4.40), 37.3 (±3.65), 24.5 (±2.51), and 27.3 (±3.02) kg/m^2^, respectively. Groups were homogeneous regarding salivary flow and the total mean amount of protein recovered (µg) both for SS and USA, showing no difference in these parameters (Table 1).

**Figure 1:**
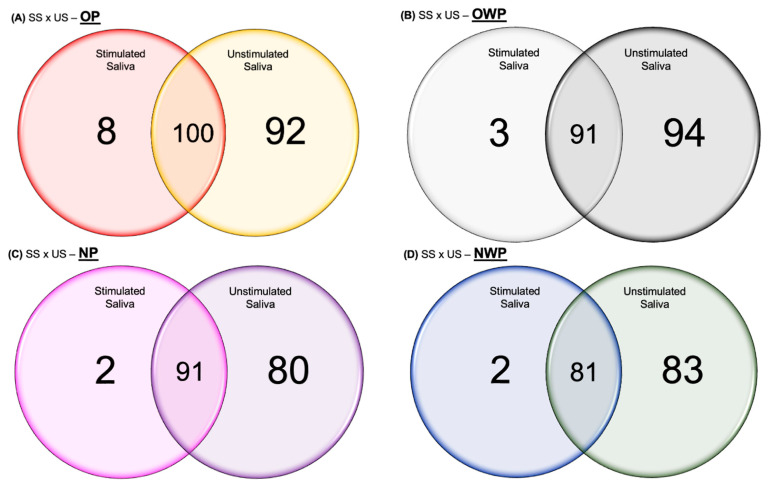
Venn diagrams showing the proteins identified in common between SS and US samples, as well as the number of proteins identified exclusively in SS and US for OP (**A**), OWP (**B**), NP (**C**), and NWP (**D**).

For the quantitative proteomic analysis, in the comparison between SS and US samples from the OP group, the total number of proteins identified was 108 and 192, respectively, among which 100 proteins were common to both types of saliva (Figure 1A). Eight proteins were identified exclusively in SS, while 92 proteins were uniquely identified in US (Table S1). Regarding the differentially expressed proteins in the OP group, 37 and 47 proteins were increased and decreased, respectively, in SS (Table S1). Among the main up-regulated proteins in SS were *Alpha-1-antitrypsin* (increased 5-fold); *Neutrophil defensin 3* (increased 4-fold); *Matrix metalloproteinase-9*; *Histatin-3* (increased 3-fold); 2 *isoforms of Basic salivary proline-rich protein*; 2 isoforms of *POTE ankyrin domain*; *Carbonic anhydrase 6*; *Actin, alpha cardiac muscle 1*; and 2 isoforms of *Hemoglobin* (increased 2-fold). Among the main down-regulated proteins in SS were *LINE-1 type transposase domain-containing protein 1* (decreased 13-fold); *Haptoglobin-related protein* (decreased 10-fold); 3 isoforms of *Hemoglobin* (decreased 8-fold); *Immunoglobulin kappa light chain*; *Haptoglobin* (decreased 5-fold); *Deleted in malignant brain tumors 1 protein*; *Immunoglobulin heavy constant gamma 2*; *Glucose-6-phosphate isomerase* (decreased 4-fold); *Lactoperoxidase*; *Protein S100-A9*; *Transaldolase*; *Alpha-2-macroglobulin*; *Immunoglobulin heavy constant gamma 4* (decreased 3-fold); *Protein S100-A8*; and *Thioredoxin* (decreased almost 3-fold) (Table 2).

**Figure 2:**
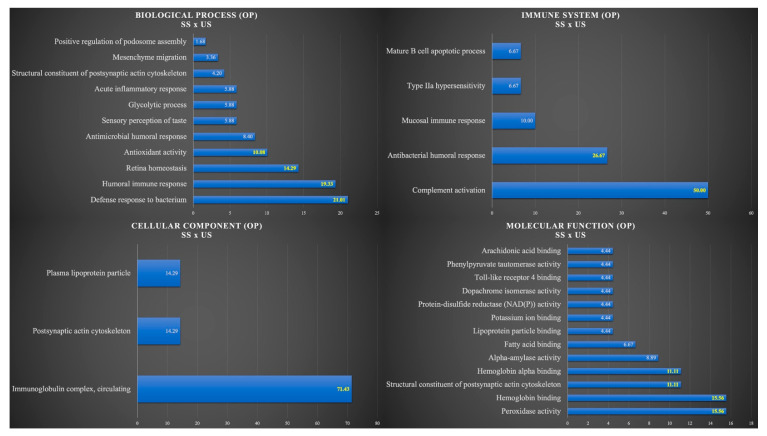
Functional analysis of the distribution of proteins identified with differential expression between SS and US in OP group.

Figure 2 shows the functional analysis for the comparison between SS and US in the OP group. Among them, we would like to highlight the categories with the highest percentages of genes in the biological process and immune system. For the biological process, the categories were defense response to a bacterium (21.01%), humoral immune response (19.33%), retina homeostasis (14.29%), and antioxidant activity (10.08%). For the immune system, the categories were complement activation (50%) and antibacterial humoral response (26.67%) (Figure 2).

In the comparison between SS and US from the OWP group, the total number of proteins identified was 94 and 185, respectively, among which 91 proteins were common to both types of saliva (Figure 1B). Three proteins were identified exclusively in SS, while ninety-four proteins were uniquely identified in US (Table S2). Regarding the differentially expressed proteins in the OWP group, 14 and 55 proteins were increased and decreased, respectively, in SS (Table S2). Among the main up-regulated proteins in SS were *Statherin* (increased 18-fold); 2 isoforms of *Immunoglobulin* (increased 3-fold); *Salivary acidic proline-rich phosphoprotein ½* (increased 3-fold); and *Histatin-3* (increased almost 3-fold). Among the main down-regulated proteins in SS were *Transaldolase; Profilin-1* (decreased 10-fold); *Myeloblastin* (decreased 7-fold); *6-phosphogluconate dehydrogenase, decarboxylating* (decreased 6-fold); *Pyruvate kinase PKM* (decreased 5-fold); 2 isoforms of *Neutrophil defensin*; 2 isoforms of *Basic salivary proline-rich protein* (decreased 4-fold); 7 isoforms of *Immunoglobulin*; *Glyceraldehyde-3-phosphate dehydrogenase*; 2 isoforms of *Protein S100*; *Lysozyme C* (decreased 3-fold) (Table 3).

**Figure 3:**
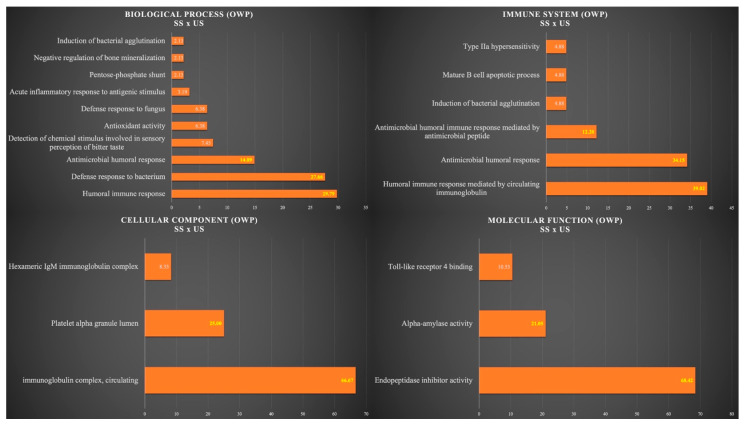
Functional analysis of the distribution of proteins identified with differential expression between SS and US in OWP group.

Figure 3 shows the functional analysis for the comparison between SS and US in the OWP group. Among them, we would like to highlight the categories with the highest percentages of genes in the biological process and immune system. For the biological process, the categories were humoral immune response (29.79%), defense response to bacterium (27.66%), and antimicrobial humoral response (14.89%). For the immune system, the categories were humoral immune response mediated by circulating immunoglobulin (39.02%), antimicrobial humoral response (34.15%), and antimicrobial humoral immune response mediated by antimicrobial peptide (12.20%) (Figure 3).

In the comparison between SS and US from the NP group, the total number of proteins identified was 93 and 171, respectively, among which 91 proteins were common to both types of saliva (Figure 1C). Two proteins were identified exclusively in SS, while eighty proteins were uniquely identified in US (Table S3). Regarding the differentially expressed proteins in the NP group, 36 and 42 proteins were increased and decreased, respectively, in SS (Table S3). Among the main up-regulated proteins in SS were *Haptoglobin* (increased 9-fold); 2 isoforms of *Basic salivary proline-rich protein*; *Histatin-3*; *Alpha-1-antitrypsin*; *Apolipoprotein A-I* (increased 4-fold); *Hemopexin*; *Cystatin-B* (increased 3-fold); and *BPI fold-containing family B member 2* (increased almost 3-fold). Among the main down-regulated proteins in SS were *Lysozyme C* (decreased 15-fold); *Protein S100-A9* (decreased 7-fold); *Beta-actin-like protein 2*; *Proline-rich protein 4*; *Mucin-7* (decreased 5-fold); 2 isoforms of *Immunoglobulin*; *Hemoglobin subunit delta* (decreased 3-fold) (Table 4).

**Figure 4:**
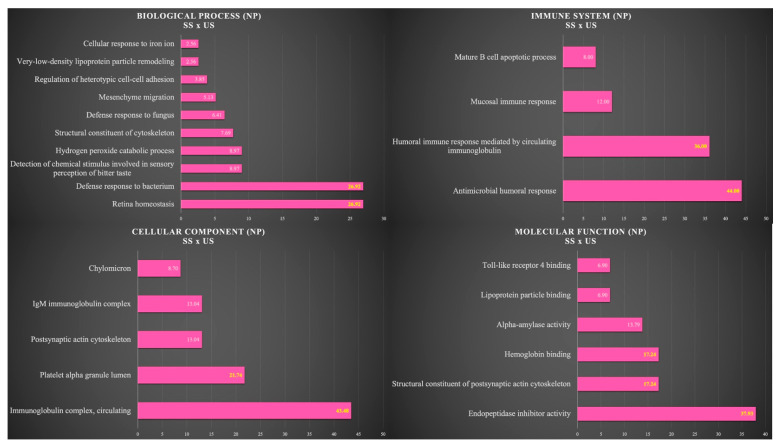
Functional analysis of the distribution of proteins identified with differential expression between SS and US in NP group.

Figure 4 shows the functional analysis for the comparison between SS and US in the NP group. Among them, we would like to highlight the categories with the highest percentages of genes in the biological process and immune system. For the biological process, the categories were retina homeostasis (26.92%) and defense response to bacterium (26.92%). For the immune system, the categories were antimicrobial humoral response (44%) and humoral immune response mediated by circulating immunoglobulin (36%) (Figure 4).

In the comparison between SS and US from the NWP group, the total number of proteins identified was 83 and 164, respectively, among which 81 proteins were common to both types of saliva (Figure 1D). Two proteins were identified exclusively in SS, while eighty-three proteins were uniquely identified in US (Table S4). Regarding the differentially expressed proteins in the NWP group, 18 and 48 proteins were increased and decreased, respectively, in SS (Table S4). Among the main up-regulated proteins in SS were *Submaxillary gland androgen-regulated protein 3B*; *Beta-2-microglobulin* (increased almost 3-fold); *Protein S100-A9*; *Mucin-7*; *Immunoglobulin lambda-1 light chain* (increased 2-fold). Among the main down-regulated proteins in SS were *Putative lipocalin 1-like protein 1* (decreased 10-fold); *Lipocalin-1* (decreased 9-fold); *Carbonic anhydrase 6* (decreased almost 4-fold); 3 isoforms of *POTE ankyrin domain*; and 3 isoforms of *Immunoglobulin* (Table 5).

**Figure 5:**
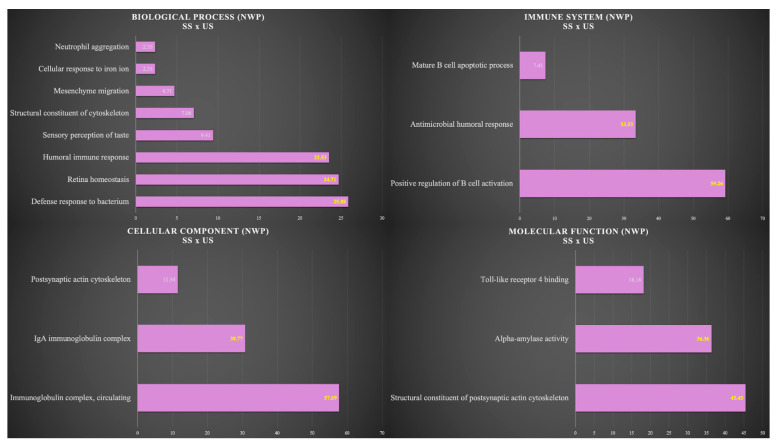
Functional analysis of the distribution of proteins identified with differential expression between SS and US in NWP group.

Figure 5 shows the functional analysis for the comparison between SS and US in the NWP group. Among them, we would like to highlight the categories with the highest percentages of genes in the biological process and immune system. For the biological process, the categories were defense response to bacterium (25.88%), retina homeostasis (24.71%), and humoral immune response (23.53%). For the immune system, the categories were positive regulation of B cell activation (59.26%) and antimicrobial humoral response (33.33%) (Figure 5).

**Figure 6:**
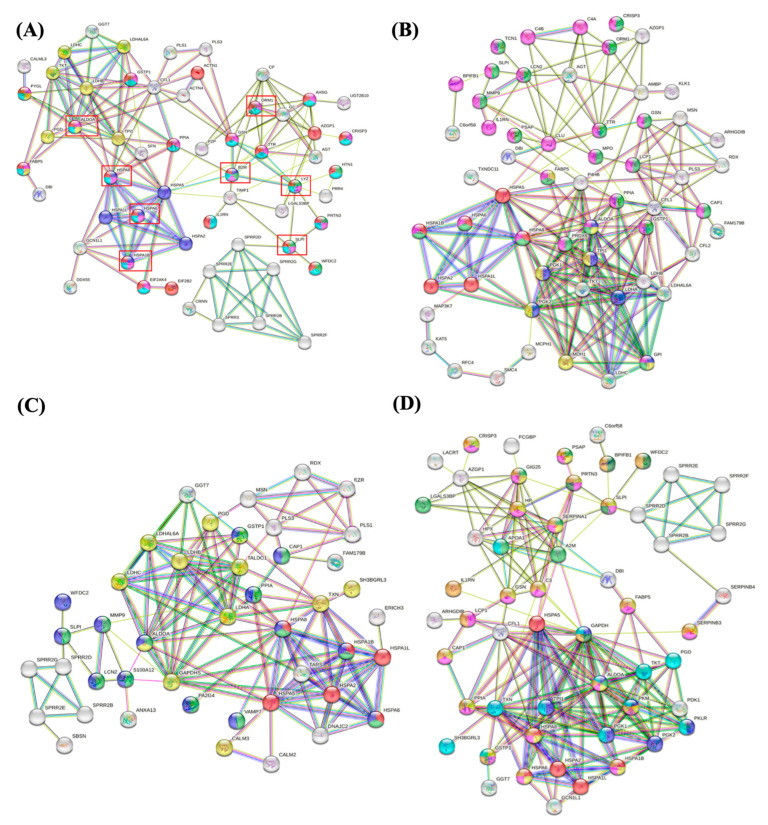
Interaction networks among unique proteins identified in US from OP (**A**), OWP (**B**), NP (**C**), and NWP (**D**).

Figure 6 shows the interaction networks among unique proteins identified in US from OP (Figure 6A), OWP (Figure 6B), NP (Figure 6C), and NWP (Figure 6D). In the OP group, dark blue nodes were related to protein refolding; green nodes were related to antimicrobial humoral response; pink, light blue, and red nodes were related to leukocyte activation involved in immune response, immune response, and immune system process, respectively; yellow nodes were related to carbohydrate metabolic process (Figure 6A). In the OWP group, red nodes were related to protein refold; dark blue and yellow nodes were related to glycolytic and glucose metabolic process, respectively; and green and pink nodes were related to leukocyte activation involved in immune response and immune response, respectively (Figure 6B). In the NP group, red nodes were related to protein refolding; green nodes were related to neutrophil degranulation; yellow nodes were related to the oxidation-reduction process; and dark blue nodes were related to immune response (Figure 6C). In the NWP group, red nodes were related to protein refolding; dark and light blue nodes were related to glycolytic and oxidation-reduction processes, respectively; dark green, pink, orange, and yellow nodes were related to antimicrobial humoral response, leukocyte activation involved in immune response, immune response, and neutrophil degranulation, respectively; and light green nodes were related to platelet degranulation (Figure 6D).

## 4. Discussion

The reduction in salivary flow in patients with obesity [10], periodontitis [11], and during the third trimester of pregnancy [12] was previously reported. Considering that collecting stimulated instead of unstimulated saliva would facilitate clinical practice, both due to the reduced collection time and the ease of obtaining an adequate amount for analysis, we sought to underly the proteomic profile of stimulated and unstimulated saliva samples from pregnant women with/without obesity and periodontitis. Our findings highlighted significant alterations in the proteomic profile of saliva when adopting different collection methods. In general, saliva stimulation decreased important proteins involved with immune response and inflammation process in all groups. Unstimulated salivary samples seem to be the best choice for the proteomic approach in pregnant women. Therefore, the null hypothesis of this study was rejected.

The proinflammatory cytokines derived from adipocytes and macrophages accumulated in the adipose tissue in obese patients can negatively affect the function of the salivary glands due to low-grade chronic inflammation in the gland [26], explaining the association between obesity and low salivary flow. Similarly, the plausible mechanisms explaining the association between low salivary flow rate and periodontitis might be due to also the increased proinflammatory cytokine levels, such as IL-2, IL-17, and TNF, triggering glandular damage and hyposalivation. Furthermore, these inflammatory cytokines accelerate the production of ROS, which, in turn, might cause structural changes in salivary gland tissue as those that occur during physiological aging [26].

As reported by Golatowski et al. (2013), the determination of the variability in whole saliva proteome is a prerequisite for the development of saliva as a diagnostic and/or prognostic human biomarker fluid. In that study, authors compared volume, protein concentrations, and proteome profile among saliva samples collected by drooling, Salivette^®^, and paraffin gum [27]. Our findings are in line with that study since an expected difference was found regarding the volume and flow rate among SS ad US samples, but no difference was found in protein concentrations. Golatowski et al. highlighted that passive drooling, paraffin gum, and Salivette^®^ each allow similar coverage of the whole saliva proteome, but the specific proteins observed depended on the collection approach [27]. In this study, specific proteins related to the immune process and inflammatory response were found mainly in unstimulated saliva samples for all groups. It is important to highlight that our main objective here was to compare stimulated and unstimulated saliva samples for each group, with no intergroup comparison for quantitative proteomic analysis, and these results were discussed in detail below.

Among the proteins elevated or exclusively present in SS samples of this study, only *Alpha-amylase 1C* and *Pancreatic alpha-amylase* were similar in all groups (Tables S1–S4). Among the proteins that were decreased in SS or exclusively present in US samples, 29 proteins were similar in all groups, such as the following: *6-phosphogluconate dehydrogenase, decarboxylating*; *Acyl-CoA-binding protein*; *Alpha-2-macroglobulin-like protein 1*; *Antileukoproteinase*; *Cysteine-rich secretory protein 3*; *Endoplasmic reticulum chaperone BiP*; *Fructose-bisphosphate aldolase A*; *Glutathione S-transferase P*; 6 isoforms of *Heat shock protein* (*Heat shock 70 kDa protein 1-like*, *Heat shock 70 kDa protein 1A*, *1B*, and *6*, *Heat shock cognate 71 kDa protein*, and *Putative heat shock 70 kDa protein 7*); 8 isoforms of *Immunoglobulin* (*Immunoglobulin alpha-2 heavy chain*, *Immunoglobulin gamma-1 heavy chain*, *Immunoglobulin heavy constant alpha 1* and *2*, *Immunoglobulin heavy constant gamma 1*, *2*, *3*, and *4*); *Lactoperoxidase*; *Lysozyme C*; *Polymeric immunoglobulin receptor*; *Protein LEG1 homolog*; *SH3 domain-binding glutamic acid-rich-like protein 3*; *Thioredoxin*; and *Transaldolase*.

These proteins are, in general, involved with the immune response process, antioxidant activity, and retina homeostasis (i.e., *Antileukoproteinase; Lysozyme C*; *Alpha-2-macroglobulin-like protein 1*; *Heat shock proteins—70 kDa 1-like*, *1A*, *1B*, *6*; *Heat shock-related 70 kDa protein 2*; *Putative Heat shock 70 kDa protein 7*; *Heat shock cognate 71 kDa*), showing that saliva stimulation decreased important proteins involved with immune response and inflammation process in all groups. Here, special attention should be given to *Antileukoproteinase* and *Lysozyme C*. *Antileukoproteinase* modulates the inflammatory and immune responses after bacterial infection and down-regulates responses to bacterial lipopolysaccharide (LPS) (UNIPROT). Similarly, *Lysozyme C* increases the activity of immunoagents through the monocyte-macrophage system by acting primarily as a bacteriolytic agent in tissues and bodily fluids. Our findings are in accordance with a recent study that compared the proteomic profile among stimulated and unstimulated saliva samples from head and neck cancer patients treated by radiotherapy [28]. In that study, authors reported that proteins involved with apoptosis, antibacterial, and acid-resistance were decreased in stimulated saliva in comparison to unstimulated saliva, indicating that unstimulated salivary flow seems to be the best alternative to search for biomarkers.

Other proteins that were also reduced in SS samples or exclusively present in US in all groups included *6-phosphogluconate dehydrogenase*, *decarboxylating*; *Fructose-bisphosphate aldolase A*; and *Transaldolase* (Tables S1–S4). Thus, if only stimulated saliva samples were analyzed, there would be a lack of understanding related to the carbohydrate metabolic process and glycolytic and glucose metabolic processes in which these proteins are involved. In addition to its important role in glycolysis and gluconeogenesis, *Fructose-bisphosphate aldolase A* has recently been found to have non-glycolytic functions, such as binding to host cell receptors to promote invasion or activating plasminogen to possibly modify host hemostasis and improve survival [29]. *Transaldolase*, in turn, is important for the balance of metabolites in the pentose-phosphate pathway, and its involvement in oxidative stress and apoptosis, in multiple sclerosis, and in cancer has been discussed [30]. Additionally, *6-phosphogluconate dehydrogenase*, *decarboxylating* catalyzes the oxidative decarboxylation of 6-phosphogluconate to ribulose 5-phosphate and CO_2_, with concomitant reduction of NADP to NADPH. In addition to these proteins, other proteins related to the metabolic process were also reduced or absent in samples of stimulated saliva in the groups of pregnant women with obesity (OP and OWP), such as the following: *Transketolase*; *Pyruvate kinase*; *Glucose-6-phosphate isomerase*; *Triosephosphate isomerase*; *L-lactate dehydrogenase A-like 6A*; *L-lactate dehydrogenase C chain*; and *Fatty acid-binding protein* (Tables S1 and S2). Therefore, in the aforementioned context, unstimulated saliva collection seems also to be a better protocol for proteomic analysis in pregnant women.

In this study, there also were some proteins decreased or absent in SS samples and in common only in periodontitis cases (OP and NP groups), for instance, *ATP-dependent RNA helicase DDX55*; 12 isoforms of *Immunoglobulin* (*Immunoglobulin heavy constant mu*, *Immunoglobulin heavy variable 3-23*, *3-30*, *3-30-3*, *3-30-5*, *3-33*, *3-53*, *3-66*, *3-74*, *Immunoglobulin kappa variable 3-11*, *3D-11*, *Immunoglobulin mu heavy chain*); *Mucin-2*; *Plastin-1*; and *Submaxillary gland androgen-regulated protein 3B* (Tables S1 and S3). We would especially like to highlight *Plastin-1*, *Mucin-2*, and *Submaxillary gland androgen-regulated protein 3B*. *Plastin-1* was an example of a protein exclusively identified in US samples from periodontitis groups (OP and NP). As we mentioned in our recent study [14], to the best of our knowledge, there has never been any proof linking *Plastin-1* with periodontitis. Recently, it was discovered that *Plastin-1* and *Plastin-3*, whose mutations cause X-linked osteoporosis, are extremely similar. Furthermore, it was discovered that *Plastin-1* regulates intracellular Ca^2+^ to encourage osteoblast development [31]. In our earlier study [14], we proposed that the existence of *Plastin-1* in pregnant women with periodontitis may represent a compensation mechanism for the high levels of inflammation and bone loss, functioning to maintain bone homeostasis. *Mucin-2* and *Submaxillary gland androgen-regulated protein 3B* are associated with periodontal diseases since the first provides a protective, lubricating barrier against particles and infectious agents at mucosal surfaces (UNIPROT), while the latter seems to bind to lipopolysaccharide of *P. gingivalis*, acting in promoting angiogenesis and establishing microvasculature [32]. Therefore, these proteins are potential biomarkers of periodontitis in pregnant women that are elevated or exclusively present in US, corroborating our previously discussed findings that point out that unstimulated saliva collection should be a preferable protocol for proteomic analysis.

It is also important to discuss our results focusing on pregnant women affected by obesity and periodontitis due to the combination of the two inflammatory conditions being of great interest nowadays. In this study, some proteins were elevated in the stimulated saliva of pregnant women from the OP group. Among them, *Alpha-1-antitrypsin*, *Neutrophil defensin 1* and *3*, *Lactotransferrin*, *MMP9*, and *Histatin-3* were involved in the immune effector process. Nevertheless, a higher number of proteins related to not only immune and inflammatory processes but also to the metabolic process were elevated or exclusively present in unstimulated saliva samples from OP. Among proteins involved in the immune process, *S100A8*, *S100A9*, *BPI fold-containing family A member 2*, *BPI fold containing family B member 2*, *Haptoglobin*, *Deleted in malignant brain tumors 1 protein*, *Lactoperoxidase*, and *Complement C3* might be considered antimicrobial agents strongly related to bacterium defense that were elevated in US samples from OP (Table S1); while *Heat shock 70 kDa protein 1B*, *2*, and *6*; *Heat shock cognate 71 kDa protein*; *Alpha-1-acid glycoprotein 1*; *Beta-2-microglobulin*; *Lysozyme C*; and *Antileukoproteinase* were examples of proteins exclusively expressed in US samples (Figure 6; Table S1). We call special attention to the importance of *Protein S100-A8*, *Heat shock 70 kDa protein 2* and *6*, *Heat shock 71 kDa protein*, and *Haptoglobin*, which were elevated or exclusively present in US samples, since these proteins were found to be potential biomarkers of the association between obesity and periodontitis during pregnancy in our previous study [14].

*Proteins S100-A8* and *S100-A9* present proinflammatory, antimicrobial, oxidant-scavenging, and apoptosis-inducing activities. Their proinflammatory activity includes recruitment of leukocytes, promotion of cytokine and chemokine production, and regulation of leukocyte adhesion and migration [33,34,35]. Regarding *Heat shock proteins* (HSPs), they are associated with the body’s metabolic and catabolic processes in addition to immune response and oxidative stress. HSP70 prevents tissue degradation by acting as a protective factor and inhibiting apoptosis [36]. Previous research indicated that HSPs were positively expressed in the periodontal pockets’ basal layer, indicating that there was an increase in the infiltration of mononuclear inflammatory cells beneath the basal layer [36,37,38]. As a result, periodontal bacteria induce the expression of HSPs in the periodontal cells, which in turn induces the production of proinflammatory cytokines by macrophages and other inflammatory cells, a mechanism that contributes to the destruction of tissue in periodontitis cases [36,37,38]. *Haptoglobin* has anti-inflammatory and antioxidative properties, acting as a bacteriostatic agent and, indirectly, as an antioxidant. Higher levels of *Haptoglobin* were associated with host defense in periodontitis cases [39,40]. Thus, these potential biomarkers of obesity plus periodontitis during pregnancy were also dependent on the type of salivary collection. Here, the unstimulated saliva samples also seem to be the best choice for the proteomic approach.

This study has some limitations. Future evaluations should be performed through different gestational trimesters, with various follow-ups after delivery, to better understand the changes in salivary proteomic profile over time, both in stimulated and unstimulated saliva samples. Ideally, future longitudinal studies with larger samples should analyze specific proteins expressed in saliva considering the different stages of periodontal diseases (including gingivitis as well) to ensure a better biological understanding regarding the progression of the diseases. Despite the limitations, to the best of our understanding, this is the first study to map the proteomic profile and to understand the differences among stimulated and unstimulated saliva samples from pregnant women with/without obesity and periodontitis, using an individual label-free quantitative shotgun proteomic analysis. The results of this study contribute to researchers around the world as it demonstrates the best saliva collection protocol that should be adopted for proteomic analysis. Furthermore, our results suggest further investigation of salivary biomarkers. The identification of biomarkers can contribute to the early diagnosis of diseases, favoring public health policies in disease prevention and health promotion since this would reduce the costs that health managers have with the sectors of greater technological density that are necessary for the rehabilitation of diseases in more advanced stages. The development of these health policies aims to achieve universal health coverage for all and to ensure people receive equitable health care and dental services.

## 5. Conclusions

In conclusion, there were significant differences in the proteomic profile of SS and US samples from pregnant women with/without obesity and periodontitis. Saliva stimulation decreased (or made absent) important proteins involved with immune response and inflammatory process in all groups. Unstimulated salivary samples seem to be the best choice for proteomic analysis in pregnant women. Our findings contribute in an unprecedented way to understanding the differences in the salivary proteomic profile of different flows in pregnant women with/without obesity and periodontitis, suggesting that future studies adopting the proteomic approach in the same target population should focus on the unstimulated salivary analysis.

## Figures and Tables

**Table 1:** Salivary and periodontal parameters of the sample.

	OP (*n* = 10) Mean ± SD Median [1st–3rd Quartiles]	OWP (*n* = 10) Mean ± SD Median [1st–3rd Quartiles]	NP (*n* = 10) Mean ± SD Median [1st–3rd Quartiles]	NWP (*n* = 10) Mean ± SD Median [1st–3rd Quartiles]	*p*
**SSF (mL/min)**	1.64 ± 0.15	1.73 ± 0.14	1.67 ± 0.14	1.76 ± 0.12	0.236 *
**Total protein SSF (ug/ptn)**	35.04 ± 14.15	30.95 ± 20.23	27.08 ± 10.30	28.65 ± 13.90	0.668 *
**USF (mL/min)**	0.56 ± 0.09	0.55 ± 0.08	0.57 ± 0.08	0.56 ± 0.06	0.426 *
**Total protein USF (ug/ptn)**	32.36 ± 15.59	36.22 ± 20.09	34.21 ± 9.86	30.44 ± 15.73	0.668 *
**PPD (mm)**	2.65 [2.45–2.81] A	2.04 [1.96–2.10] B	2.56 [2.42–2.79] A	2.02 [2.00–2.16] B	**<0.001 ^†^**
**CAL (mm)**	2.66 [2.45–2.82] A	2.07 [2.02–2.10] B	2.58 [2.44–2.79] A	2.03 [2.00–2.16] B	**<0.001 ^†^**
**Periodontitis—*n* (%)** **Stage II** **Stage III**	5 (50%) 5 (50%)	-	9 (90%) 1 (10%)	-	**<0.001 ^†^**

OP, obesity and periodontitis; OWP, obesity without periodontitis; NP, normal BMI and periodontitis; NWP, normal BMI without periodontitis; SD, standard deviation; *p*, significance level; SSF, stimulated salivary flow; USF, unstimulated salivary flow; PPD, probing pocket depth; CAL, clinical attachment level; * ANOVA; ^†^ Kruskal–Wallis (post hoc test: Dunn). Different letters indicate a statistically significant difference (*p* < 0.05). Bold values indicate significance level lower than 5%.

**Table 2:** Main expression differences (up- or down-regulated proteins by more than 2-fold) identified in stimulated saliva (SS) and unstimulated saliva (US) of pregnant women with obesity and periodontitis (OP).

Accession Number	Protein Name	Gene	Score	Fold Change	Log (e)	SD	*p*	ED
**P01009**	*Alpha-1-antitrypsin*	*SERPINA1*	69	5.87	1.77	0.06	**<0.01**	↑
**P59666**	*Neutrophil defensin 3*	*DEFA3*	903	4.85	1.58	0.18	**<0.01**	↑
**P14780**	*Matrix metalloproteinase-9*	*MMP9*	58	3.86	1.35	0.12	**<0.01**	↑
**P15516**	*Histatin-3*	*HTN3*	4616	3.78	1.33	0.09	**<0.01**	↑
**P02812**	*Basic salivary proline-rich protein 2*	*PRB2*	1417	2.72	1	0.05	**<0.01**	↑
**P04280**	*Basic salivary proline-rich protein 1*	*PRB1*	1417	2.59	0.95	0.07	**<0.01**	↑
**Q6S8J3**	*POTE ankyrin domain family member E*	*POTEE*	1087	2.39	0.87	0.07	**<0.01**	↑
**A5A3E0**	*POTE ankyrin domain family member F*	*POTEF*	1087	2.34	0.85	0.05	**<0.01**	↑
**P23280**	*Carbonic anhydrase 6*	*CA6*	1598	2.29	0.83	0.09	**<0.01**	↑
**P68032**	*Actin* *, alpha cardiac muscle 1*	*ACTC1*	2203	2.12	0.75	0.04	**<0.01**	↑
**P02042**	*Hemoglobin subunit delta*	*HBD*	138	2.10	0.74	0.06	**<0.01**	↑
**P69905**	*Hemoglobin subunit alpha*	*HBA1; HBA2*	5146	2.05	0.72	0.07	**<0.01**	↑
**Q8N4F0**	*BPI fold-containing family B member 2*	*BPIFB2*	232	0.49	−0.72	0.17	**<0.01**	↓
**P01834**	*Immunoglobulin kappa constant*	*IGKC*	5120	0.49	−0.72	0.07	**<0.01**	↓
**P01860**	*Immunoglobulin heavy constant gamma 3*	*IGHG3*	981	0.48	−0.73	0.13	**<0.01**	↓
**P01833**	*Polymeric immunoglobulin receptor*	*PIGR*	5222	0.47	−0.76	0.02	**<0.01**	↓
**P01024**	*Complement C3*	*C3*	129	0.45	−0.8	0.12	**<0.01**	↓
**P02787**	*Serotransferrin*	*TF*	1492	0.43	−0.85	0.02	**<0.01**	↓
**P0DOX6**	*Immunoglobulin mu heavy chain*	*IGM*	674	0.42	−0.87	0.09	**<0.01**	↓
**P01591**	*Immunoglobulin J chain*	*JCHAIN*	5085	0.42	−0.87	0.04	**<0.01**	↓
**P01871**	*Immunoglobulin heavy constant mu*	*IGHM*	692	0.41	−0.89	0.11	**<0.01**	↓
**P68871**	*Hemoglobin subunit beta*	*HBB*	414	0.40	−0.91	0.03	**<0.01**	↓
**P14618**	*Pyruvate kinase PKM*	*PKM*	119	0.40	−0.91	0.09	**<0.01**	↓
**P30613**	*Pyruvate kinase PKLR*	*PKLR*	80	0.39	−0.95	0.27	**0.02**	↓
**P0DOX2**	*Immunoglobulin alpha-2 heavy chain*	*IGA2*	6336	0.38	−0.96	0.02	**<0.01**	↓
**P10599**	*Thioredoxin*	*TXN*	934	0.35	−1.04	0.15	**<0.01**	↓
**P05109**	*Protein S100-A8*	*S100A8*	1531	0.35	−1.06	0.03	**<0.01**	↓
**P01861**	*Immunoglobulin heavy constant gamma 4*	*IGHG4*	701	0.33	−1.11	0.17	**<0.01**	↓
**P01023**	*Alpha-2-macroglobulin*	*A2M*	186	0.31	−1.17	0.05	**<0.01**	↓
**P37837**	*Transaldolase*	*TALDO1*	89	0.30	−1.19	0.1	**<0.01**	↓
**P06702**	*Protein S100-A9*	*S100A9*	555	0.28	−1.29	0.03	**<0.01**	↓
**P22079**	*Lactoperoxidase*	*LPO*	546	0.27	−1.3	0.12	**<0.01**	↓
**P06744**	*Glucose-6-phosphate isomerase*	*GPI*	134	0.23	−1.46	0.11	**<0.01**	↓
**P01859**	*Immunoglobulin heavy constant gamma 2*	*IGHG2*	680	0.23	−1.47	0.08	**<0.01**	↓
**Q9UGM3**	*Deleted in malignant brain tumors 1 protein*	*DMBT1*	365	0.23	−1.49	0.09	**<0.01**	↓
**P00738**	*Haptoglobin*	*HP*	1526	0.19	−1.65	0.07	**<0.01**	↓
**P0DOX7**	*Immunoglobulin kappa light chain*	*IGK*	2435	0.17	−1.77	0.04	**<0.01**	↓
**P69892**	*Hemoglobin subunit gamma-2*	*HBG2*	408	0.12	−2.15	0.03	**<0.01**	↓
**P69891**	*Hemoglobin subunit gamma-1*	*HBG1*	408	0.11	−2.17	0.03	**<0.01**	↓
**P02100**	*Hemoglobin subunit epsilon*	*HBE1*	408	0.11	−2.18	0.03	**<0.01**	↓
**P00739**	*Haptoglobin-related protein*	*HPR*	280	0.10	−2.32	0.09	**<0.01**	↓
**Q5T7N2**	*LINE-1 type transposase domain-containing protein 1*	*L1TD1*	35	0.08	−2.59	0.03	**0.01**	↓

Note: Log (e) (“e” is a constant = 2.71); SD, standard deviation; *p*, statistical significance (adjusted by False Discovery Rate - FDR = 4); ED, Expression differences; ↑ = up-regulated in SS (1-*p* > 0.95); ↓ = down-regulated in SS (*p* < 0.05). Bold values indicate significance level lower than 5%.

**Table 3:** Main expression differences (up- or down-regulated proteins by more than 2-fold) identified in stimulated saliva (SS) and unstimulated saliva (US) of pregnant women with obesity but without periodontitis (OWP).

Accession Number	Protein Name	Gene	Score	Fold Change	Log (e)	SD	*p*	ED
**P02808**	*Statherin*	*STATH*	23,896	18.73	2.93	0.14	**<0.01**	↑
**P01871**	*Immunoglobulin heavy constant mu*	*IGHM*	379	3.42	1.23	0.06	**<0.01**	↑
**P0DOX6**	*Immunoglobulin mu heavy chain*	*IGM*	373	3.25	1.18	0.09	**<0.01**	↑
**P02810**	*Salivary acidic proline-rich phosphoprotein 1/2*	*PRH1; PRH2*	1111	3.22	1.17	0.02	**<0.01**	↑
**P15516**	*Histatin-3*	*HTN3*	1165	2.89	1.06	0.1	**<0.01**	↑
**P01860**	*Immunoglobulin heavy constant gamma 3*	*IGHG3*	108	0.49	−0.71	0.08	**<0.01**	↓
**P01591**	*Immunoglobulin J chain*	*JCHAIN*	1073	0.47	−0.76	0.06	**<0.01**	↓
**P01876**	*Immunoglobulin heavy constant alpha 1*	*IGHA1*	1734	0.46	−0.77	0.02	**<0.01**	↓
**P02788**	*Lactotransferrin*	*LTF*	1044	0.43	−0.85	0.07	**<0.01**	↓
**P69905**	*Hemoglobin subunit alpha*	*HBA1; HBA2*	80	0.40	−0.91	0.07	**<0.01**	↓
**P12273**	*Prolactin-inducible protein*	*PIP*	3571	0.38	−0.98	0.02	**<0.01**	↓
**P10599**	*Thioredoxin*	*TXN*	461	0.37	−0.99	0.24	**0.01**	↓
**P05109**	*Protein S100-A8*	*S100A8*	5810	0.34	−1.08	0.04	**<0.01**	↓
**P61626**	*Lysozyme C*	*LYZ*	3669	0.31	−1.16	0.04	**<0.01**	↓
**P04406**	*Glyceraldehyde-3-phosphate dehydrogenase*	*GAPDH*	250	0.31	−1.18	0.1	**<0.01**	↓
**P0DOY2**	*Immunoglobulin lambda constant 2*	*IGLC2*	201	0.29	−1.24	0.04	**<0.01**	↓
**P59665**	*Neutrophil defensin 1*	*DEFA1; DEFA1B*	2405	0.26	−1.34	0.2	**<0.01**	↓
**P0DOX2**	*Immunoglobulin alpha-2 heavy chain*	*IGA2*	1386	0.26	−1.35	0.01	**<0.01**	↓
**P04280**	*Basic salivary proline-rich protein 1*	*PRB1*	85	0.25	−1.4	0.02	**<0.01**	↓
**P02812**	*Basic salivary proline-rich protein 2*	*PRB2*	85	0.25	−1.4	0.03	**<0.01**	↓
**P0CG04**	*Immunoglobulin lambda constant 1*	*IGLC1*	115	0.24	−1.43	0.04	**<0.01**	↓
**P01877**	*Immunoglobulin heavy constant alpha 2*	*IGHA2*	1437	0.23	−1.45	0.01	**<0.01**	↓
**P59666**	*Neutrophil defensin 3*	*DEFA3*	2405	0.23	−1.45	0.19	**<0.01**	↓
**P06702**	*Protein S100-A9*	*S100A9*	1584	0.20	−1.63	0.06	**<0.01**	↓
**P14618**	*Pyruvate kinase PKM*	*PKM*	118	0.18	−1.74	0.11	**<0.01**	↓
**P52209**	*6-phosphogluconate dehydrogenase,* *decarboxylating*	*PGD*	159	0.16	−1.85	0.15	**<0.01**	↓
**P24158**	*Myeloblastin*	*PRTN3*	379	0.13	−2.05	0.08	**<0.01**	↓
**P07737**	*Profilin-1*	*PFN1*	449	0.10	−2.32	0.06	**<0.01**	↓
**P37837**	*Transaldolase*	*TALDO1*	203	0.10	−2.35	0.07	**<0.01**	↓

Note: Log (e) (“e” is a constant = 2.71); SD, standard deviation; *p*, statistical significance (adjusted by False Discovery Rate - FDR = 4); ED, Expression differences; ↑ = up-regulated in SS (1-*p* > 0.95); ↓ = down-regulated in SS (*p* < 0.05). Bold values indicate significance level lower than 5%.

**Table 4:** Main expression differences (up- or down-regulated proteins by more than 2-fold) identified in stimulated saliva (SS) and unstimulated saliva (US) of pregnant women with normal BMI but with periodontitis (NP).

Accession Number	Protein Name	Gene	Score	Fold Change	Log (e)	SD	*p*	ED
**P00738**	*Haptoglobin*	*HP*	114	9.68	2.27	0.06	**<0.01**	↑
**P04280**	*Basic salivary proline-rich protein 1*	*PRB1*	366	5.26	1.66	0.25	**<0.01**	↑
**P15516**	*Histatin-3*	*HTN3*	1825	4.90	1.59	0.4	**<0.01**	↑
**P01009**	*Alpha-1-antitrypsin*	*SERPINA1*	180	4.71	1.55	0.08	**<0.01**	↑
**P02812**	*Basic salivary proline-rich protein 2*	*PRB2*	366	4.48	1.5	0.05	**<0.01**	↑
**P02647**	*Apolipoprotein A-I*	*APOA1*	4432	4.10	1.41	0.05	**<0.01**	↑
**P02790**	*Hemopexin*	*HPX*	153	3.25	1.18	0.05	**<0.01**	↑
**P04080**	*Cystatin-B*	*CSTB*	1239	3.16	1.15	0.08	**<0.01**	↑
**Q8N4F0**	*BPI fold-containing family B member 2*	*BPIFB2*	422	2.77	1.02	0.06	**<0.01**	↑
**P02810**	*Salivary acidic proline-rich phosphoprotein 1/2*	*PRH1; PRH2*	2308	2.51	0.92	0.16	**<0.01**	↑
**P69892**	*Hemoglobin subunit gamma-2*	*HBG2*	271	2.39	0.87	0.13	**0.01**	↑
**P69891**	*Hemoglobin subunit gamma-1*	*HBG1*	271	2.39	0.87	0.12	**<0.01**	↑
**P02100**	*Hemoglobin subunit epsilon*	*HBE1*	271	2.39	0.87	0.12	**<0.01**	↑
**P02808**	*Statherin*	*STATH*	2286	2.27	0.82	0.22	**<0.01**	↑
**P02679**	*Fibrinogen gamma chain*	*FGG*	118	2.23	0.8	0.13	**<0.01**	↑
**P59666**	*Neutrophil defensin 3*	*DEFA3*	1062	2.12	0.75	0.06	**<0.01**	↑
**P20742**	*Pregnancy zone protein*	*PZP*	212	2.10	0.74	0.09	**<0.01**	↑
**P59665**	*Neutrophil defensin 1*	*DEFA1; DEFA1B*	1062	2.10	0.74	0.06	**<0.01**	↑
**P0DTE8**	*Alpha-amylase 1C*	*AMY1C*	3994	2.08	0.73	0.01	**<0.01**	↑
**P22079**	*Lactoperoxidase*	*LPO*	82	0.49	−0.72	0.11	**<0.01**	↓
**P0CG38**	*POTE ankyrin domain family member I*	*POTEI*	257	0.47	−0.75	0.07	**<0.01**	↓
**P02788**	*Lactotransferrin*	*LTF*	58	0.47	−0.75	0.26	**0.01**	↓
**P0CG39**	*POTE ankyrin domain family member J*	*POTEJ*	176	0.47	−0.76	0.1	**<0.01**	↓
**P68133**	*Actin, alpha skeletal muscle*	*ACTA1*	904	0.45	−0.8	0.07	**<0.01**	↓
**Q8NHQ9**	*ATP-dependent RNA helicase DDX55*	*DDX55*	169	0.44	−0.82	0.26	**0.02**	↓
**P63267**	*Actin, gamma-enteric smooth muscle*	*ACTG2*	904	0.43	−0.85	0.07	**<0.01**	↓
**Q5VSP4**	*Putative lipocalin 1-like protein 1*	*LCN1P1*	967	0.43	−0.85	0.08	**<0.01**	↓
**Q6S8J3**	*POTE ankyrin domain family member E*	*POTEE*	366	0.39	−0.94	0.05	**<0.01**	↓
**A5A3E0**	*POTE ankyrin domain family member F*	*POTEF*	366	0.39	−0.95	0.06	**<0.01**	↓
**P68032**	*Actin, alpha cardiac muscle 1*	*ACTC1*	904	0.37	−1	0.04	**<0.01**	↓
**Q9BYX7**	*Putative beta-actin-like protein 3*	*POTEKP*	109	0.36	−1.02	0.05	**<0.01**	↓
**P62736**	*Actin, aortic smooth muscle*	*ACTA2*	904	0.35	−1.05	0.05	**<0.01**	↓
**P63261**	*Actin, cytoplasmic 2*	*ACTG1*	1384	0.34	−1.09	0.02	**<0.01**	↓
**P60709**	*Actin, cytoplasmic 1*	*ACTB*	1384	0.33	−1.1	0.02	**<0.01**	↓
**P02042**	*Hemoglobin subunit delta*	*HBD*	499	0.32	−1.13	0.05	**<0.01**	↓
**A0M8Q6**	*Immunoglobulin lambda constant 7*	*IGLC7*	320	0.23	−1.48	0.08	**<0.01**	↓
**Q8TAX7**	*Mucin-7*	*MUC7*	842	0.20	−1.63	0.02	**<0.01**	↓
**Q16378**	*Proline-rich protein 4*	*PRR4*	984	0.18	−1.69	0.08	**0.01**	↓
**Q562R1**	*Beta-actin-like protein 2*	*ACTBL2*	362	0.17	−1.75	0.02	**<0.01**	↓
**P01859**	*Immunoglobulin heavy constant gamma 2*	*IGHG2*	85	0.17	−1.8	0.06	**<0.01**	↓
**P06702**	*Protein S100-A9*	*S100A9*	344	0.13	−2.02	0.04	**<0.01**	↓
**P61626**	*Lysozyme C*	*LYZ*	3223	0.06	−2.77	0.03	**<0.01**	↓

Note: Log (e) (“e” is a constant = 2.71); SD, standard deviation; *p*, statistical significance (adjusted by False Discovery Rate - FDR = 4); ED, Expression differences; ↑ = up-regulated in SS (1-*p* > 0.95); ↓ = down-regulated in SS (*p* < 0.05). Bold values indicate significance level lower than 5%.

**Table 5:** Main expression differences (up- or down-regulated proteins by more than 2-fold) identified in stimulated saliva (SS) and unstimulated saliva (US) of pregnant women with normal BMI and without periodontitis (NWP).

Accession Number	Protein Name	Gene	Score	Fold Change	Log (e)	SD	*p*	ED
**P02814**	*Submaxillary gland androgen-regulated protein 3B*	*SMR3B*	51,408	2.94	1.08	0.02	**<0.01**	↑
**P61769**	*Beta-2-microglobulin*	*B2M*	132	2.94	1.08	0.1	**<0.01**	↑
**P06702**	*Protein S100-A9*	*S100A9*	181	2.53	0.93	0.08	**<0.01**	↑
**Q8TAX7**	*Mucin-7*	*MUC7*	1497	2.27	0.82	0.04	**<0.01**	↑
**P0DOX8**	*Immunoglobulin lambda-1 light chain*	*IGL1*	3256	2.08	0.73	0.06	**<0.01**	↑
**Q9BYX7**	*Putative beta-actin-like protein 3*	*POTEKP*	254	0.50	−0.7	0.09	**<0.01**	↓
**P01036**	*Cystatin-S*	*CST4*	16,480	0.50	−0.7	0.02	**<0.01**	↓
**A0M8Q6**	*Immunoglobulin lambda constant 7*	*IGLC7*	2152	0.48	−0.74	0.23	**<0.01**	↓
**P07737**	*Profilin-1*	*PFN1*	859	0.46	−0.77	0.16	**<0.01**	↓
**P61626**	*Lysozyme C*	*LYZ*	3743	0.45	−0.8	0.16	**<0.01**	↓
**P02810**	*Salivary acidic proline-rich phosphoprotein 1/2*	*PRH1; PRH2*	3931	0.43	−0.85	0.02	**<0.01**	↓
**Q562R1**	*Beta-actin-like protein 2*	*ACTBL2*	626	0.43	−0.85	0.09	**<0.01**	↓
**A5A3E0**	*POTE ankyrin domain family member F*	*POTEF*	337	0.42	−0.86	0.07	**<0.01**	↓
**P37837**	*Transaldolase*	*TALDO1*	199	0.42	−0.87	0.23	**<0.01**	↓
**Q96DA0**	*Zymogen granule protein 16 homolog B*	*ZG16B*	22,215	0.42	−0.87	0.03	**<0.01**	↓
**Q6S8J3**	*POTE ankyrin domain family member E*	*POTEE*	337	0.41	−0.88	0.05	**<0.01**	↓
**P0CG38**	*POTE ankyrin domain family member I*	*POTEI*	83	0.36	−1.02	0.09	**<0.01**	↓
**P01861**	*Immunoglobulin heavy constant gamma 4*	*IGHG4*	122	0.32	−1.13	0.29	**<0.01**	↓
**P01859**	*Immunoglobulin heavy constant gamma 2*	*IGHG2*	30	0.31	−1.17	0.29	**<0.01**	↓
**P0CG39**	*POTE ankyrin domain family member J*	*POTEJ*	83	0.30	−1.22	0.17	**<0.01**	↓
**P23280**	*Carbonic anhydrase 6*	*CA6*	1177	0.25	−1.38	0.03	**<0.01**	↓
**P31025**	*Lipocalin-1*	*LCN1*	5807	0.10	−2.3	0.03	**<0.01**	↓
**Q5VSP4**	*Putative lipocalin 1-like protein 1*	*LCN1P1*	4592	0.10	−2.33	0.03	**<0.01**	↓

Note: Log (e) (“e” is a constant = 2.71); SD, standard deviation; *p*, statistical significance (adjusted by False Discovery Rate - FDR = 4); ED, Expression differences; ↑ = up-regulated in SS (1-*p* > 0.95); ↓ = down-regulated in SS (*p* < 0.05). Bold values indicate significance level lower than 5%.

## Data Availability

Data presented in this study are available in the article and Supplementary Materials and made available upon request to the corresponding authors. The mass spectrometric proteomic data have been deposited to the ProteomeXchange Consortium via the PRIDE partner repository with the data set identifier PXD040373.

## Supplementary Materials

The following supporting information can be downloaded at: https://www.mdpi.com/article/10.3390/cells12101389/s1, Table S1: Expression differences and unique proteins identified in stimulated saliva (SS) and unstimulated saliva (US) of pregnant women with obesity and periodontitis (OP); Table S2: Expression differences and unique proteins identified in stimulated saliva (SS) and unstimulated saliva (US) of pregnant women with obesity but without periodontitis (OWP); Table S3: Expression differences and unique proteins identified in stimulated saliva (SS) and unstimulated saliva (US) of pregnant women with normal BMI but with periodontitis (NP); Table S4: Expression differences and unique proteins identified in stimulated saliva (SS) and unstimulated saliva (US) of pregnant women with normal BMI and without periodontitis (NWP).
