# Supplementary material for: Is There a Difference in the Proteomic Profile of Stimulated and Unstimulated Saliva Samples from Pregnant Women with/without Obesity and Periodontitis?
Source: Cells. 2023 May 14;12(10):1389. doi: 10.3390/cells12101389 (PMC10216649; doi:10.3390/cells12101389)
Supplement: Supplementary file 1 [file cells-12-01389-s001.zip › cells-2289259-supplementary/Supplemetary files/Supplementary file 1.pdf]

Table S1. Expression differences and unique proteins identified in stimulated saliva (SS) and unstimulated saliva (US) of pregnant women with obesity and periodontitis (OP)

Accession number	Protein name	Gene	Score	Fold change	Log(e)	SD	*p*	ED
**P01009**	***Alpha-1-antitrypsin***	***SERPINA1***	**69**	**5.87**	**1.77**	**0.06**	**< 0.01**	↑
**P59666**	***Neutrophil defensin 3***	***DEFA3***	**903**	**4.85**	**1.58**	**0.18**	**< 0.01**	↑
**P14780**	***Matrix metalloproteinase-9***	***MMP9***	**58**	**3.86**	**1.35**	**0.12**	**< 0.01**	↑
**P15516**	***Histatin-3***	***HTN3***	**4616**	**3.78**	**1.33**	**0.09**	**< 0.01**	↑
**P02812**	***Basic salivary proline-rich protein 2***	***PRB2***	**1417**	**2.72**	**1**	**0.05**	**< 0.01**	↑
**P04280**	***Basic salivary proline-rich protein 1***	***PRB1***	**1417**	**2.59**	**0.95**	**0.07**	**< 0.01**	↑
**Q6S8J3**	***POTE ankyrin domain family member E***	***POTEE***	**1087**	**2.39**	**0.87**	**0.07**	**< 0.01**	↑
**A5A3E0**	***POTE ankyrin domain family member F***	***POTEF***	**1087**	**2.34**	**0.85**	**0.05**	**< 0.01**	↑
**P23280**	***Carbonic anhydrase 6***	***CA6***	**1598**	**2.29**	**0.83**	**0.09**	**< 0.01**	↑
**P68032**	***Actin, alpha cardiac muscle 1***	***ACTC1***	**2203**	**2.12**	**0.75**	**0.04**	**< 0.01**	↑
**P02042**	***Hemoglobin subunit delta***	***HBD***	**138**	**2.10**	**0.74**	**0.06**	**< 0.01**	↑
**P69905**	***Hemoglobin subunit alpha***	***HBA1; HBA2***	**5146**	**2.05**	**0.72**	**0.07**	**< 0.01**	↑
Q9BYX7	*Putative beta-actin-like protein 3*	*POTEKP*	586	1.99	0.69	0.08	< 0.01	↑
Q5VSP4	*Putative lipocalin 1-like protein 1*	*LCN1P1*	1750	1.95	0.67	0.08	< 0.01	↑
Q562R1	*Beta-actin-like protein 2*	*ACTBL2*	1347	1.88	0.63	0.04	< 0.01	↑
P60709	*Actin, cytoplasmic 1*	*ACTB*	3189	1.82	0.6	0.03	< 0.01	↑
P63261	*Actin, cytoplasmic 2*	*ACTG1*	3189	1.77	0.57	0.04	< 0.01	↑
P02679	*Fibrinogen gamma chain*	*FGG*	209	1.70	0.53	0.26	< 0.01	↑
Q96DA0	*Zymogen granule protein 16 homolog B*	*ZG16B*	5726	1.68	0.52	0.03	< 0.01	↑
P00558	*Phosphoglycerate kinase 1*	*PGK1*	38	1.65	0.5	0.18	< 0.01	↑
P02788	*Lactotransferrin*	*LTF*	185	1.60	0.47	0.07	< 0.01	↑
P02647	*Apolipoprotein A-I*	*APOA1*	1035	1.55	0.44	0.1	< 0.01	↑
P59665	*Neutrophil defensin 1*	*DEFA1; DEFA1B*	903	1.52	0.42	0.07	< 0.01	↑
P09228	*Cystatin-SA*	*CST2*	14286	1.51	0.41	0.03	< 0.01	↑
P00338	*L-lactate dehydrogenase A chain*	*LDHA*	126	1.45	0.37	0.14	0.03	↑
P13796	*Plastin-2*	*LCP1*	66	1.31	0.27	0.09	0.01	↑
P04746	*Pancreatic alpha-amylase*	*AMY2A*	42667	1.30	0.26	0.01	< 0.01	↑
P68133	*Actin, alpha skeletal muscle*	*ACTA1*	2203	1.27	0.24	0.04	< 0.01	↑
P12273	*Prolactin-inducible protein*	*PIP*	17911	1.27	0.24	0.05	< 0.01	↑
P63267	*Actin, gamma-enteric smooth muscle*	*ACTG2*	2203	1.26	0.23	0.04	< 0.01	↑
P62736	*Actin, aortic smooth muscle*	*ACTA2*	2203	1.26	0.23	0.04	< 0.01	↑
P04406	*Glyceraldehyde-3-phosphate dehydrogenase*	*GAPDH*	327	1.25	0.22	0.12	0.04	↑
P01036	*Cystatin-S*	*CST4*	42785	1.14	0.13	0.02	< 0.01	↑
P02768	*Albumin*	*ALB*	15911	1.11	0.1	0.02	< 0.01	↑
P19961	*Alpha-amylase 2B*	*AMY2B*	47202	1.08	0.08	0.01	< 0.01	↑
P0DUB6	*Alpha-amylase 1A*	*AMY1A*	59213	1.04	0.04	0.01	< 0.01	↑
P0DTE8	*Alpha-amylase 1C*	*AMY1C*	59213	1.04	0.04	0.01	< 0.01	↑
P0DTE7	*Alpha-amylase 1B*	*AMY1B*	59213	0.92	-0.08	0.01	< 0.01	↓
P26038	*Moesin*	*MSN*	45	0.70	-0.35	0.12	0.01	↓

Q96DR5	*BPI fold-containing family A member 2*	*BPIFA2*	1792	0.68	-0.38	0.07	< 0.01	↓
P0DMV8	*Heat shock 70 kDa protein 1A*	*HSPA1A*	70	0.65	-0.43	0.12	< 0.01	↓
P31025	*Lipocalin-1*	*LCN1*	2326	0.63	-0.47	0.06	< 0.01	↓
P0DOY3	*Immunoglobulin lambda constant 3*	*IGLC3*	1937	0.61	-0.5	0.05	< 0.01	↓
B9A064	*Immunoglobulin lambda-like polypeptide 5*	*IGLL5*	1143	0.59	-0.52	0.06	< 0.01	↓
P0CF74	*Immunoglobulin lambda constant 6*	*IGLC6*	1728	0.59	-0.52	0.06	< 0.01	↓
P0CG04	*Immunoglobulin lambda constant 1*	*IGLC1*	1143	0.59	-0.52	0.06	< 0.01	↓
P13929	*Beta-enolase*	*ENO3*	29	0.59	-0.52	0.14	< 0.01	↓
P0DOX8	*Immunoglobulin lambda-1 light chain*	*IGL1*	1143	0.59	-0.53	0.07	< 0.01	↓
P01876	*Immunoglobulin heavy constant alpha 1*	*IGHA1*	11765	0.57	-0.56	0.01	< 0.01	↓
P0DOY2	*Immunoglobulin lambda constant 2*	*IGLC2*	1937	0.57	-0.57	0.04	< 0.01	↓
P01877	*Immunoglobulin heavy constant alpha 2*	*IGHA2*	6656	0.57	-0.57	0.01	< 0.01	↓
P02814	*Submaxillary gland androgen-regulated protein 3B*	*SMR3B*	49706	0.52	-0.66	0.01	< 0.01	↓
P01857	*Immunoglobulin heavy constant gamma 1*	*IGHG1*	1966	0.51	-0.67	0.04	< 0.01	↓
P0DOX5	*Immunoglobulin gamma-1 heavy chain*	*IGG1*	1966	0.51	-0.68	0.03	< 0.01	↓
**Q8N4F0**	***BPI fold-containing family B member 2***	***BPIFB2***	**232**	**0.49**	**-0.72**	**0.17**	**< 0.01**	↓
**P01834**	***Immunoglobulin kappa constant***	***IGKC***	**5120**	**0.49**	**-0.72**	**0.07**	**< 0.01**	↓
**P01860**	***Immunoglobulin heavy constant gamma 3***	***IGHG3***	**981**	**0.48**	**-0.73**	**0.13**	**< 0.01**	↓
**P01833**	***Polymeric immunoglobulin receptor***	***PIGR***	**5222**	**0.47**	**-0.76**	**0.02**	**< 0.01**	↓
**P01024**	***Complement C3***	***C3***	**129**	**0.45**	**-0.8**	**0.12**	**< 0.01**	↓
**P02787**	***Serotransferrin***	***TF***	**1492**	**0.43**	**-0.85**	**0.02**	**< 0.01**	↓
**P0DOX6**	***Immunoglobulin mu heavy chain***	***IGM***	**674**	**0.42**	**-0.87**	**0.09**	**< 0.01**	↓
**P01591**	***Immunoglobulin J chain***	***JCHAIN***	**5085**	**0.42**	**-0.87**	**0.04**	**< 0.01**	↓
**P01871**	***Immunoglobulin heavy constant mu***	***IGHM***	**692**	**0.41**	**-0.89**	**0.11**	**< 0.01**	↓
**P68871**	***Hemoglobin subunit beta***	***HBB***	**414**	**0.40**	**-0.91**	**0.03**	**< 0.01**	↓
**P14618**	***Pyruvate kinase PKM***	***PKM***	**119**	**0.40**	**-0.91**	**0.09**	**< 0.01**	↓
**P30613**	***Pyruvate kinase PKLR***	***PKLR***	**80**	**0.39**	**-0.95**	**0.27**	**0.02**	↓
**P0DOX2**	***Immunoglobulin alpha-2 heavy chain***	***IGA2***	**6336**	**0.38**	**-0.96**	**0.02**	**< 0.01**	↓
**P10599**	***Thioredoxin***	***TXN***	**934**	**0.35**	**-1.04**	**0.15**	**< 0.01**	↓
**P05109**	***Protein S100-A8***	***S100A8***	**1531**	**0.35**	**-1.06**	**0.03**	**< 0.01**	↓
**P01861**	***Immunoglobulin heavy constant gamma 4***	***IGHG4***	**701**	**0.33**	**-1.11**	**0.17**	**< 0.01**	↓
**P01023**	***Alpha-2-macroglobulin***	***A2M***	**186**	**0.31**	**-1.17**	**0.05**	**< 0.01**	↓
**P37837**	***Transaldolase***	***TALDO1***	**89**	**0.30**	**-1.19**	**0.1**	**< 0.01**	↓
**P06702**	***Protein S100-A9***	***S100A9***	**555**	**0.28**	**-1.29**	**0.03**	**< 0.01**	↓
**P22079**	***Lactoperoxidase***	***LPO***	**546**	**0.27**	**-1.3**	**0.12**	**< 0.01**	↓
**P06744**	***Glucose-6-phosphate isomerase***	***GPI***	**134**	**0.23**	**-1.46**	**0.11**	**< 0.01**	↓
**P01859**	***Immunoglobulin heavy constant gamma 2***	***IGHG2***	**680**	**0.23**	**-1.47**	**0.08**	**< 0.01**	↓
**Q9UGM3**	***Deleted in malignant brain tumors 1 protein***	***DMBT1***	**365**	**0.23**	**-1.49**	**0.09**	**< 0.01**	↓
**P00738**	***Haptoglobin***	***HP***	**1526**	**0.19**	**-1.65**	**0.07**	**< 0.01**	↓
**P0DOX7**	***Immunoglobulin kappa light chain***	***IGK***	**2435**	**0.17**	**-1.77**	**0.04**	**< 0.01**	↓
**P69892**	***Hemoglobin subunit gamma-2***	***HBG2***	**408**	**0.12**	**-2.15**	**0.03**	**< 0.01**	↓
**P69891**	***Hemoglobin subunit gamma-1***	***HBG1***	**408**	**0.11**	**-2.17**	**0.03**	**< 0.01**	↓
**P02100**	***Hemoglobin subunit epsilon***	***HBE1***	**408**	**0.11**	**-2.18**	**0.03**	**< 0.01**	↓
**P00739**	***Haptoglobin-related protein***	***HPR***	**280**	**0.10**	**-2.32**	**0.09**	**< 0.01**	↓
**Q5T7N2**	***LINE-1 type transposase domain-containing protein 1***	***L1TD1***	**35**	**0.08**	**-2.59**	**0.03**	**0.01**	↓

P00915	*Carbonic anhydrase 1*	*CA1*	262	SS	SS	SS	SS	SS
P02008	*Hemoglobin subunit zeta*	*HBZ*	141	SS	SS	SS	SS	SS
Q06830	*Peroxiredoxin-1*	*PRDX1*	131	SS	SS	SS	SS	SS
P32119	*Peroxiredoxin-2*	*PRDX2*	224	SS	SS	SS	SS	SS
Q14693	*Phosphatidate phosphatase LPIN1*	*LPIN1*	30	SS	SS	SS	SS	SS
P06703	*Protein S100-A6*	*S100A6*	351	SS	SS	SS	SS	SS
Q15276	*Rab GTPase-binding effector protein 1*	*RABEP1*	24	SS	SS	SS	SS	SS
P11684	*Uteroglobin*	*SCGB1A1*	865	SS	SS	SS	SS	SS
P31947	*14-3-3 protein sigma*	*SFN*	448	US	US	US	US	US
P52209	*6-phosphogluconate dehydrogenase, decarboxylating*	*PGD*	139	US	US	US	US	US
P07108	*Acyl-CoA-binding protein*	*DBI*	826	US	US	US	US	US
Q6P587	*Acylpyruvase FAHD1, mitochondrial*	*FAHD1*	568	US	US	US	US	US
P02763	*Alpha-1-acid glycoprotein 1*	*ORM1*	280	US	US	US	US	US
P02765	*Alpha-2-HS-glycoprotein*	*AHSG*	427	US	US	US	US	US
A8K2U0	*Alpha-2-macroglobulin-like protein 1*	*A2ML1*	138	US	US	US	US	US
P12814	*Alpha-actinin-1*	*ACTN1*	62	US	US	US	US	US
O43707	*Alpha-actinin-4*	*ACTN4*	50	US	US	US	US	US
P01019	*Angiotensinogen*	*AGT*	180	US	US	US	US	US
P03973	*Antileukoproteinase*	*SLPI*	1114	US	US	US	US	US
Q96LR9	*Apolipoprotein L domain-containing protein 1*	*APOLD1*	70	US	US	US	US	US
Q8NHQ9	*ATP-dependent RNA helicase DDX55*	*DDX55*	141	US	US	US	US	US
P61769	*Beta-2-microglobulin*	*B2M*	396	US	US	US	US	US
P27482	*Calmodulin-like protein 3*	*CALML3*	227	US	US	US	US	US
P00450	*Ceruloplasmin*	*CP*	590	US	US	US	US	US
P23528	*Cofilin-1*	*CFL1*	895	US	US	US	US	US
Q9UBG3	*Cornulin*	*CRNN*	224	US	US	US	US	US
P28325	*Cystatin-D*	*CST5*	580	US	US	US	US	US
P54108	*Cysteine-rich secretory protein 3*	*CRISP3*	159	US	US	US	US	US
Q92616	*eIF-2-alpha kinase activator GCN1*	*GCN1*	45	US	US	US	US	US
Q9P2K8	*eIF-2-alpha kinase GCN2*	*EIF2AK4*	306	US	US	US	US	US
P11021	*Endoplasmic reticulum chaperone BiP*	*HSPA5*	34	US	US	US	US	US
Q01469	*Fatty acid-binding protein 5*	*FABP5*	769	US	US	US	US	US
Q5W0V3	*FHF complex subunit HOOK interacting protein 2A*	*FHIP2A*	152	US	US	US	US	US
P04075	*Fructose-bisphosphate aldolase A*	*ALDOA*	127	US	US	US	US	US
Q08380	*Galectin-3-binding protein*	*LGALS3BP*	99	US	US	US	US	US
P06396	*Gelsolin*	*GSN*	110	US	US	US	US	US
Q9UJ14	*Glutathione hydrolase 7*	*GGT7*	114	US	US	US	US	US
P09211	*Glutathione S-transferase P*	*GSTP1*	280	US	US	US	US	US
P06737	*Glycogen phosphorylase, liver form*	*PYGL*	45	US	US	US	US	US
P34931	*Heat shock 70 kDa protein 1-like*	*HSPA1L*	68	US	US	US	US	US
P0DMV9	*Heat shock 70 kDa protein 1B*	*HSPA1B*	68	US	US	US	US	US
P17066	*Heat shock 70 kDa protein 6*	*HSPA6*	31	US	US	US	US	US
P11142	*Heat shock cognate 71 kDa protein*	*HSPA8*	34	US	US	US	US	US
P54652	*Heat shock-related 70 kDa protein 2*	*HSPA2*	34	US	US	US	US	US
P15515	*Histatin-1*	*HTN1*	3253	US	US	US	US	US

P01764	*Immunoglobulin heavy variable 3-23*	*IGHV3-23*	458	US	US	US	US	US
P01768	*Immunoglobulin heavy variable 3-30*	*IGHV3-30*	458	US	US	US	US	US
P0DP02	*Immunoglobulin heavy variable 3-30-3*	*IGHV3-30-3*	458	US	US	US	US	US
P0DP03	*Immunoglobulin heavy variable 3-30-5*	*IGHV3-30-5*	458	US	US	US	US	US
P01772	*Immunoglobulin heavy variable 3-33*	*IGHV3-33*	458	US	US	US	US	US
P01767	*Immunoglobulin heavy variable 3-53*	*IGHV3-53*	458	US	US	US	US	US
A0A0C4DH42	*Immunoglobulin heavy variable 3-66*	*IGHV3-66*	458	US	US	US	US	US
A0A0B4J1X5	*Immunoglobulin heavy variable 3-74*	*IGHV3-74*	458	US	US	US	US	US
P04433	*Immunoglobulin kappa variable 3-11*	*IGKV3-11*	692	US	US	US	US	US
A0A0A0MRZ8	*Immunoglobulin kappa variable 3D-11*	*IGKV3D-11*	692	US	US	US	US	US
Q8WYH8	*Inhibitor of growth protein 5*	*ING5*	568	US	US	US	US	US
P18510	*Interleukin-1 receptor antagonist protein*	*IL1RN*	112	US	US	US	US	US
P06870	*Kallikrein-1*	*KLK1*	95	US	US	US	US	US
Q6ZMR3	*L-lactate dehydrogenase A-like 6A*	*LDHAL6A*	24	US	US	US	US	US
P07195	*L-lactate dehydrogenase B chain*	*LDHB*	24	US	US	US	US	US
P07864	*L-lactate dehydrogenase C chain*	*LDHC*	24	US	US	US	US	US
P61626	*Lysozyme C*	*LYZ*	391	US	US	US	US	US
P01033	*Metalloproteinase inhibitor 1*	*TIMP1*	175	US	US	US	US	US
Q02817	*Mucin-2*	*MUC2*	45	US	US	US	US	US
Q8NCY6	*Myb/SANT-like DNA-binding domain-containing protein 4*	*MSANTD4*	165	US	US	US	US	US
P24158	*Myeloblastin*	*PRTN3*	87	US	US	US	US	US
P80303	*Nucleobindin-2*	*NUCB2*	79	US	US	US	US	US
Q8NGQ2	*Olfactory receptor 6Q1*	*OR6Q1*	201	US	US	US	US	US
P62937	*Peptidyl-prolyl cis-trans isomerase A*	*PPIA*	725	US	US	US	US	US
Q9Y536	*Peptidyl-prolyl cis-trans isomerase A-like 4A*	*PPIAL4A*	130	US	US	US	US	US
Q14651	*Plastin-1*	*PLS1*	88	US	US	US	US	US
P13797	*Plastin-3*	*PLS3*	34	US	US	US	US	US
P20742	*Pregnancy zone protein*	*PZP*	33	US	US	US	US	US
Q16378	*Proline-rich protein 4*	*PRR4*	80	US	US	US	US	US
Q8N6L0	*Protein KASH5*	*KASH5*	62	US	US	US	US	US
Q6P5S2	*Protein LEG1 homolog*	*LEG1*	2785	US	US	US	US	US
Q9Y5F8	*Protocadherin gamma-B7*	*PCDHGB7*	47	US	US	US	US	US
P48741	*Putative heat shock 70 kDa protein 7*	*HSPA7*	31	US	US	US	US	US
P50120	*Retinol-binding protein 2*	*RBP2*	390	US	US	US	US	US
P52566	*Rho GDP-dissociation inhibitor 2*	*ARHGDIB*	201	US	US	US	US	US
Q9H299	*SH3 domain-binding glutamic acid-rich-like protein 3*	*SH3BGRL3*	282	US	US	US	US	US
P35326	*Small proline-rich protein 2A*	*SPRR2A*	711	US	US	US	US	US
P35325	*Small proline-rich protein 2B*	*SPRR2B*	950	US	US	US	US	US
P22532	*Small proline-rich protein 2D*	*SPRR2D*	950	US	US	US	US	US
P22531	*Small proline-rich protein 2E*	*SPRR2E*	781	US	US	US	US	US
Q96RM1	*Small proline-rich protein 2F*	*SPRR2F*	243	US	US	US	US	US
Q9BYE4	*Small proline-rich protein 2G*	*SPRR2G*	539	US	US	US	US	US
Q9UBC9	*Small proline-rich protein 3*	*SPRR3*	900	US	US	US	US	US
Q14515	*SPARC-like protein 1*	*SPARCL1*	31	US	US	US	US	US
P20061	*Transcobalamin-1*	*TCN1*	81	US	US	US	US	US

P29401	*Transketolase*	*TKT*	376	US	US	US	US	US
P49770	*Translation initiation factor eIF-2B subunit beta*	*EIF2B2*	36	US	US	US	US	US
P02766	*Transthyretin*	*TTR*	452	US	US	US	US	US
P60174	*Triosephosphate isomerase*	*TPI1*	137	US	US	US	US	US
P36537	*UDP-glucuronosyltransferase 2B10*	*UGT2B10*	23	US	US	US	US	US
Q9BY64	*UDP-glucuronosyltransferase 2B28*	*UGT2B28*	23	US	US	US	US	US
P02774	*Vitamin D-binding protein*	*GC*	171	US	US	US	US	US
Q14508	*WAP four-disulfide core domain protein 2*	*WFDC2*	896	US	US	US	US	US
Q9UJU3	*Zinc finger protein 112*	*ZNF112*	47	US	US	US	US	US
P25311	*Zinc-alpha-2-glycoprotein*	*AZGP1*	210	US	US	US	US	US
P07205	*Phosphoglycerate kinase 2*	*PGK2*	19	1.23	0.21	0.26	0.14	SE
P07737	*Profilin-1*	*PFN1*	748	1.22	0.2	0.12	0.06	SE
P02790	*Hemopexin*	*HPX*	610	1.16	0.15	0.11	0.11	SE
P02810	*Salivary acidic proline-rich phosphoprotein 1/2*	*PRH1; PRH2*	2797	1.14	0.13	0.19	0.24	SE
P0CG39	*POTE ankyrin domain family member J*	*POTEJ*	374	1.07	0.07	0.07	0.16	SE
P01034	*Cystatin-C*	*CST3*	3600	1.07	0.07	0.13	0.28	SE
P80188	*Neutrophil gelatinase-associated lipocalin*	*LCN2*	387	1.04	0.04	0.1	0.37	SE
P04080	*Cystatin-B*	*CSTB*	11884	1.03	0.03	0.11	0.49	SE
A0M8Q6	*Immunoglobulin lambda constant 7*	*IGLC7*	965	1.03	0.03	0.11	0.41	SE
Q8TAX7	*Mucin-7*	*MUC7*	778	1.01	0.01	0.06	0.45	SE
P01037	*Cystatin-SN*	*CST1*	34577	0.99	-0.01	0.02	0.23	SE
P06733	*Alpha-enolase*	*ENO1*	451	0.98	-0.02	0.08	0.38	SE
P0CG38	*POTE ankyrin domain family member I*	*POTEI*	524	0.97	-0.03	0.07	0.34	SE
P09104	*Gamma-enolase*	*ENO2*	70	0.90	-0.1	0.22	0.33	SE
Q01518	*Adenylyl cyclase-associated protein 1*	*CAP1*	145	0.52	-0.66	0.33	0.05	SE
P02808	*Statherin*	*STATH*	49709	0.47	-0.75	0.83	0.3	SE

Note: Log(e) ("e" is a constant = 2.71); SD, standard deviation; *p*, statistical significance (adjusted by False Discovery Rate–FDR = 4); ↑ = up-regulated in SS (1-p > 0.95); ED, Expression differences; ↓ = down-regulated in SS ($p < 0.05$); SE = similar expression in SS and US; bold lines refer to up- or down-regulated proteins by more than 2-fold
