# Supplementary material for: Is There a Difference in the Proteomic Profile of Stimulated and Unstimulated Saliva Samples from Pregnant Women with/without Obesity and Periodontitis?
Source: Cells. 2023 May 14;12(10):1389. doi: 10.3390/cells12101389 (PMC10216649; doi:10.3390/cells12101389)
Supplement: Supplementary file 1 [file cells-12-01389-s001.zip › cells-2289259-supplementary/Supplemetary files/Supplementary file 2.pdf]

Table S2. Expression differences and unique proteins identified in stimulated saliva (SS) and unstimulated saliva (US) of pregnant women with obesity but without periodontitis (OWP)

Accession number	Protein name	Gene	Score	Fold change	Log(e)	SD	*p*	ED
**P02808**	***Statherin***	***STATH***	**23896**	**18.73**	**2.93**	**0.14**	**< 0.01**	↑
**P01871**	***Immunoglobulin heavy constant mu***	***IGHM***	**379**	**3.42**	**1.23**	**0.06**	**< 0.01**	↑
**P0DOX6**	***Immunoglobulin mu heavy chain***	***IGM***	**373**	**3.25**	**1.18**	**0.09**	**< 0.01**	↑
**P02810**	***Salivary acidic proline-rich phosphoprotein 1/2***	***PRH1; PRH2***	**1111**	**3.22**	**1.17**	**0.02**	**< 0.01**	↑
**P15516**	***Histatin-3***	***HTN3***	**1165**	**2.89**	**1.06**	**0.1**	**< 0.01**	↑
P0DUB6	*Alpha-amylase 1A*	*AMY1A*	12079	1.72	0.54	0.01	< 0.01	↑
P0DTE8	*Alpha-amylase 1C*	*AMY1C*	12079	1.72	0.54	0.01	< 0.01	↑
P0DTE7	*Alpha-amylase 1B*	*AMY1B*	12079	1.68	0.52	0.01	< 0.01	↑
Q9UBC9	*Small proline-rich protein 3*	*SPRR3*	1377	1.42	0.35	0.06	< 0.01	↑
P23280	*Carbonic anhydrase 6*	*CA6*	499	1.42	0.35	0.06	< 0.01	↑
P19961	*Alpha-amylase 2B*	*AMY2B*	10632	1.30	0.26	0.01	< 0.01	↑
P04746	*Pancreatic alpha-amylase*	*AMY2A*	8827	1.25	0.22	0.01	< 0.01	↑
P01037	*Cystatin-SN*	*CST1*	3683	1.23	0.21	0.02	< 0.01	↑
P01834	*Immunoglobulin kappa constant*	*IGKC*	343	1.17	0.16	0.03	< 0.01	↑
P31025	*Lipocalin-1*	*LCN1*	1422	0.90	-0.1	0.06	0.04	↓
P01009	*Alpha-1-antitrypsin*	*SERPINA1*	75	0.87	-0.14	0.04	< 0.01	↓
P09228	*Cystatin-SA*	*CST2*	513	0.78	-0.25	0.02	< 0.01	↓
P02675	*Fibrinogen beta chain*	*FGB*	2032	0.77	-0.26	0.08	< 0.01	↓
A0M8Q6	*Immunoglobulin lambda constant 7*	*IGLC7*	201	0.76	-0.27	0.07	< 0.01	↓
P02671	*Fibrinogen alpha chain*	*FGA*	360	0.73	-0.31	0.08	< 0.01	↓
P00450	*Ceruloplasmin*	*CP*	882	0.71	-0.34	0.11	< 0.01	↓
P02768	*Albumin*	*ALB*	3664	0.70	-0.35	0.02	< 0.01	↓
B9A064	*Immunoglobulin lambda-like polypeptide 5*	*IGLL5*	115	0.70	-0.36	0.06	< 0.01	↓
P22079	*Lactoperoxidase*	*LPO*	55	0.70	-0.36	0.11	< 0.01	↓
P0DOX8	*Immunoglobulin lambda-1 light chain*	*IGL1*	115	0.69	-0.37	0.06	< 0.01	↓
P28325	*Cystatin-D*	*CST5*	114	0.69	-0.37	0.08	< 0.01	↓
P02790	*Hemopexin*	*HPX*	125	0.64	-0.45	0.06	< 0.01	↓
P0DOY3	*Immunoglobulin lambda constant 3*	*IGLC3*	201	0.63	-0.47	0.06	< 0.01	↓
P0CF74	*Immunoglobulin lambda constant 6*	*IGLC6*	201	0.62	-0.48	0.05	< 0.01	↓
Q8TAX7	*Mucin-7*	*MUC7*	330	0.61	-0.5	0.07	< 0.01	↓
P20742	*Pregnancy zone protein*	*PZP*	25	0.60	-0.51	0.13	< 0.01	↓
P01036	*Cystatin-S*	*CST4*	4223	0.60	-0.51	0.02	< 0.01	↓
P02774	*Vitamin D-binding protein*	*GC*	1144	0.59	-0.53	0.11	< 0.01	↓
P01857	*Immunoglobulin heavy constant gamma 1*	*IGHG1*	603	0.58	-0.54	0.05	< 0.01	↓
P01024	*Complement C3*	*C3*	25	0.58	-0.55	0.06	< 0.01	↓
P01833	*Polymeric immunoglobulin receptor*	*PIGR*	2946	0.58	-0.55	0.02	< 0.01	↓
P02765	*Alpha-2-HS-glycoprotein*	*AHSG*	579	0.57	-0.57	0.24	0.03	↓
P02787	*Serotransferrin*	*TF*	207	0.55	-0.6	0.04	< 0.01	↓
P0DOX5	*Immunoglobulin gamma-1 heavy chain*	*IGG1*	603	0.55	-0.6	0.06	< 0.01	↓
P02042	*Hemoglobin subunit delta*	*HBD*	201	0.54	-0.61	0.08	< 0.01	↓

P01861	*Immunoglobulin heavy constant gamma 4*	*IGHG4*	42	0.54	-0.62	0.07	< 0.01	↓
Q5VSP4	*Putative lipocalin 1-like protein 1*	*LCN1P1*	662	0.52	-0.66	0.06	< 0.01	↓
P01023	*Alpha-2-macroglobulin*	*A2M*	44	0.51	-0.67	0.07	< 0.01	↓
P04080	*Cystatin-B*	*CSTB*	240	0.51	-0.67	0.08	< 0.01	↓
P01859	*Immunoglobulin heavy constant gamma 2*	*IGHG2*	42	0.51	-0.68	0.08	< 0.01	↓
**P01860**	***Immunoglobulin heavy constant gamma 3***	***IGHG3***	**108**	**0.49**	**-0.71**	**0.08**	**< 0.01**	↓
**P01591**	***Immunoglobulin J chain***	***JCHAIN***	**1073**	**0.47**	**-0.76**	**0.06**	**< 0.01**	↓
**P01876**	***Immunoglobulin heavy constant alpha 1***	***IGHA1***	**1734**	**0.46**	**-0.77**	**0.02**	**< 0.01**	↓
**P02788**	***Lactotransferrin***	***LTF***	**1044**	**0.43**	**-0.85**	**0.07**	**< 0.01**	↓
**P69905**	***Hemoglobin subunit alpha***	***HBA1; HBA2***	**80**	**0.40**	**-0.91**	**0.07**	**< 0.01**	↓
**P12273**	***Prolactin-inducible protein***	***PIP***	**3571**	**0.38**	**-0.98**	**0.02**	**< 0.01**	↓
**P10599**	***Thioredoxin***	***TXN***	**461**	**0.37**	**-0.99**	**0.24**	**0.01**	↓
**P05109**	***Protein S100-A8***	***S100A8***	**5810**	**0.34**	**-1.08**	**0.04**	**< 0.01**	↓
**P61626**	***Lysozyme C***	***LYZ***	**3669**	**0.31**	**-1.16**	**0.04**	**< 0.01**	↓
**P04406**	***Glyceraldehyde-3-phosphate dehydrogenase***	***GAPDH***	**250**	**0.31**	**-1.18**	**0.1**	**< 0.01**	↓
**P0DOY2**	***Immunoglobulin lambda constant 2***	***IGLC2***	**201**	**0.29**	**-1.24**	**0.04**	**< 0.01**	↓
**P59665**	***Neutrophil defensin 1***	***DEFA1; DEFA1B***	**2405**	**0.26**	**-1.34**	**0.2**	**< 0.01**	↓
**P0DOX2**	***Immunoglobulin alpha-2 heavy chain***	***IGA2***	**1386**	**0.26**	**-1.35**	**0.01**	**< 0.01**	↓
**P04280**	***Basic salivary proline-rich protein 1***	***PRB1***	**85**	**0.25**	**-1.4**	**0.02**	**< 0.01**	↓
**P02812**	***Basic salivary proline-rich protein 2***	***PRB2***	**85**	**0.25**	**-1.4**	**0.03**	**< 0.01**	↓
**P0CG04**	***Immunoglobulin lambda constant 1***	***IGLC1***	**115**	**0.24**	**-1.43**	**0.04**	**< 0.01**	↓
**P01877**	***Immunoglobulin heavy constant alpha 2***	***IGHA2***	**1437**	**0.23**	**-1.45**	**0.01**	**< 0.01**	↓
**P59666**	***Neutrophil defensin 3***	***DEFA3***	**2405**	**0.23**	**-1.45**	**0.19**	**< 0.01**	↓
**P06702**	***Protein S100-A9***	***S100A9***	**1584**	**0.20**	**-1.63**	**0.06**	**< 0.01**	↓
**P14618**	***Pyruvate kinase PKM***	***PKM***	**118**	**0.18**	**-1.74**	**0.11**	**< 0.01**	↓
**P52209**	***6-phosphogluconate dehydrogenase, decarboxylating***	***PGD***	**159**	**0.16**	**-1.85**	**0.15**	**< 0.01**	↓
**P24158**	***Myeloblastin***	***PRTN3***	**379**	**0.13**	**-2.05**	**0.08**	**< 0.01**	↓
**P07737**	***Profilin-1***	***PFN1***	**449**	**0.10**	**-2.32**	**0.06**	**< 0.01**	↓
**P37837**	***Transaldolase***	***TALDO1***	**203**	**0.10**	**-2.35**	**0.07**	**< 0.01**	↓
Q6Q4G3	*Aminopeptidase Q*	*LVRN*	24	SS	SS	SS	SS	SS
P15515	*Histatin-1*	*HTN1*	5818	SS	SS	SS	SS	SS
P11684	*Uteroglobin*	*SCGB1A1*	406	SS	SS	SS	SS	SS
P07108	*Acyl-CoA-binding protein*	*DBI*	155	US	US	US	US	US
Q01518	*Adenylyl cyclase-associated protein 1*	*CAP1*	278	US	US	US	US	US
P02763	*Alpha-1-acid glycoprotein 1*	*ORM1*	756	US	US	US	US	US
A8K2U0	*Alpha-2-macroglobulin-like protein 1*	*A2ML1*	20	US	US	US	US	US
P01019	*Angiotensinogen*	*AGT*	193	US	US	US	US	US
P03973	*Antileukoproteinase*	*SLPI*	459	US	US	US	US	US
Q8TDL5	*BPI fold-containing family B member 1*	*BPIFB1*	58	US	US	US	US	US
Q5SW79	*Centrosomal protein of 170 kDa*	*CEP170*	90	US	US	US	US	US
O15078	*Centrosomal protein of 290 kDa*	*CEP290*	162	US	US	US	US	US
P10909	*Clusterin*	*CLU*	191	US	US	US	US	US
P23528	*Cofilin-1*	*CFL1*	930	US	US	US	US	US

Q9Y281	*Cofilin-2*	*CFL2*	139	US	US	US	US	US
A2RUR9	*Coiled-coil domain-containing protein 144A*	*CCDC144A*	52	US	US	US	US	US
Q3MJ40	*Coiled-coil domain-containing protein 144B*	*CCDC144BP*	45	US	US	US	US	US
P0C0L4	*Complement C4-A*	*C4A*	72	US	US	US	US	US
P0C0L5	*Complement C4-B*	*C4B; C4B_2*	70	US	US	US	US	US
Q9UBG3	*Cornulin*	*CRNN*	100	US	US	US	US	US
P54108	*Cysteine-rich secretory protein 3*	*CRISP3*	245	US	US	US	US	US
Q02487	*Desmocollin-2*	*DSC2*	76	US	US	US	US	US
P11021	*Endoplasmic reticulum chaperone BiP*	*HSPA5*	207	US	US	US	US	US
Q01469	*Fatty acid-binding protein 5*	*FABP5*	707	US	US	US	US	US
P04075	*Fructose-bisphosphate aldolase A*	*ALDOA*	177	US	US	US	US	US
P06396	*Gelsolin*	*GSN*	272	US	US	US	US	US
P06744	*Glucose-6-phosphate isomerase*	*GPI*	436	US	US	US	US	US
P09211	*Glutathione S-transferase P*	*GSTP1*	330	US	US	US	US	US
P34931	*Heat shock 70 kDa protein 1-like*	*HSPA1L*	299	US	US	US	US	US
P0DMV8	*Heat shock 70 kDa protein 1A*	*HSPA1A*	349	US	US	US	US	US
P0DMV9	*Heat shock 70 kDa protein 1B*	*HSPA1B*	330	US	US	US	US	US
P17066	*Heat shock 70 kDa protein 6*	*HSPA6*	270	US	US	US	US	US
P11142	*Heat shock cognate 71 kDa protein*	*HSPA8*	245	US	US	US	US	US
P54652	*Heat shock-related 70 kDa protein 2*	*HSPA2*	245	US	US	US	US	US
Q92993	*Histone acetyltransferase KAT5*	*KAT5*	157	US	US	US	US	US
A0A075B6P5	*Immunoglobulin kappa variable 2-28*	*IGKV2-28*	184	US	US	US	US	US
A2NJV5	*Immunoglobulin kappa variable 2-29*	*IGKV2-29*	184	US	US	US	US	US
P06310	*Immunoglobulin kappa variable 2-30*	*IGKV2-30*	184	US	US	US	US	US
A0A087WW87	*Immunoglobulin kappa variable 2-40*	*IGKV2-40*	184	US	US	US	US	US
A0A0A0MRZ7	*Immunoglobulin kappa variable 2D-26*	*IGKV2D-26*	184	US	US	US	US	US
P01615	*Immunoglobulin kappa variable 2D-28*	*IGKV2D-28*	184	US	US	US	US	US
A0A075B6S2	*Immunoglobulin kappa variable 2D-29*	*IGKV2D-29*	184	US	US	US	US	US
A0A075B6S6	*Immunoglobulin kappa variable 2D-30*	*IGKV2D-30*	184	US	US	US	US	US
P01614	*Immunoglobulin kappa variable 2D-40*	*IGKV2D-40*	184	US	US	US	US	US
P18510	*Interleukin-1 receptor antagonist protein*	*IL1RN*	241	US	US	US	US	US
P06870	*Kallikrein-1*	*KLK1*	107	US	US	US	US	US
O14782	*Kinesin-like protein KIF3C*	*KIF3C*	52	US	US	US	US	US
P00338	*L-lactate dehydrogenase A chain*	*LDHA*	877	US	US	US	US	US
Q6ZMR3	*L-lactate dehydrogenase A-like 6A*	*LDHAL6A*	351	US	US	US	US	US
P07195	*L-lactate dehydrogenase B chain*	*LDHB*	325	US	US	US	US	US
P07864	*L-lactate dehydrogenase C chain*	*LDHC*	325	US	US	US	US	US
O95274	*Ly6/PLAUR domain-containing protein 3*	*LYPD3*	144	US	US	US	US	US
O60449	*Lymphocyte antigen 75*	*LY75*	18	US	US	US	US	US
P40925	*Malate dehydrogenase, cytoplasmic*	*MDH1*	117	US	US	US	US	US
O75556	*Mammaglobin-B*	*SCGB2A1*	522	US	US	US	US	US
P14780	*Matrix metalloproteinase-9*	*MMP9*	179	US	US	US	US	US
O00255	*Menin*	*MEN1*	47	US	US	US	US	US
Q8NEM0	*Microcephalin*	*MCPH1*	50	US	US	US	US	US
O43318	*Mitogen-activated protein kinase kinase kinase 7*	*MAP3K7*	42	US	US	US	US	US

P26038	*Moesin*	*MSN*	110	US	US	US	US	US
P05164	*Myeloperoxidase*	*MPO*	87	US	US	US	US	US
O75161	*Nephrocystin-4*	*NPHP4*	62	US	US	US	US	US
P80188	*Neutrophil gelatinase-associated lipocalin*	*LCN2*	2719	US	US	US	US	US
P62937	*Peptidyl-prolyl cis-trans isomerase A*	*PPIA*	151	US	US	US	US	US
Q9Y536	*Peptidyl-prolyl cis-trans isomerase A-like 4A*	*PPIAL4A*	40	US	US	US	US	US
P30041	*Peroxiredoxin-6*	*PRDX6*	153	US	US	US	US	US
P00558	*Phosphoglycerate kinase 1*	*PGK1*	35	US	US	US	US	US
P07205	*Phosphoglycerate kinase 2*	*PGK2*	127	US	US	US	US	US
P13796	*Plastin-2*	*LCP1*	164	US	US	US	US	US
P13797	*Plastin-3*	*PLS3*	34	US	US	US	US	US
Q8TBY8	*Polyamine-modulated factor 1-binding protein 1*	*PMFBP1*	45	US	US	US	US	US
P07602	*Prosaposin*	*PSAP*	96	US	US	US	US	US
P02760	*Protein AMBP*	*AMBP*	149	US	US	US	US	US
P07237	*Protein disulfide-isomerase*	*P4HB*	180	US	US	US	US	US
Q6P5S2	*Protein LEG1 homolog*	*LEG1*	270	US	US	US	US	US
Q6NUI1	*Putative coiled-coil domain-containing protein 144 N-terminal-like*	*CCDC144NL*	39	US	US	US	US	US
Q8IYA2	*Putative coiled-coil domain-containing protein 144C*	*CCDC144CP*	50	US	US	US	US	US
P48741	*Putative heat shock 70 kDa protein 7*	*HSPA7*	270	US	US	US	US	US
P35241	*Radixin*	*RDX*	88	US	US	US	US	US
P35249	*Replication factor C subunit 4*	*RFC4*	66	US	US	US	US	US
P52566	*Rho GDP-dissociation inhibitor 2*	*ARHGDIB*	206	US	US	US	US	US
Q8N392	*Rho GTPase-activating protein 18*	*ARHGAP18*	59	US	US	US	US	US
Q96QB1	*Rho GTPase-activating protein 7*	*DLC1*	85	US	US	US	US	US
Q9H299	*SH3 domain-binding glutamic acid-rich-like protein 3*	*SH3BGRL3*	413	US	US	US	US	US
Q9NTJ3	*Structural maintenance of chromosomes protein 4*	*SMC4*	46	US	US	US	US	US
Q6PKC3	*Thioredoxin domain-containing protein 11*	*TXNDC11*	17	US	US	US	US	US
Q9Y4F4	*TOG array regulator of axonemal microtubules protein 1*	*TOGARAM1*	52	US	US	US	US	US
P20061	*Transcobalamin-1*	*TCN1*	127	US	US	US	US	US
P29401	*Transketolase*	*TKT*	95	US	US	US	US	US
P02766	*Transthyretin*	*TTR*	255	US	US	US	US	US
P60174	*Triosephosphate isomerase*	*TPI1*	520	US	US	US	US	US
Q9BXT4	*Tudor domain-containing protein 1*	*TDRD1*	19	US	US	US	US	US
P36941	*Tumor necrosis factor receptor superfamily member 3*	*LTBR*	87	US	US	US	US	US
O94966	*Ubiquitin carboxyl-terminal hydrolase 19*	*USP19*	20	US	US	US	US	US
Q96KN7	*X-linked retinitis pigmentosa GTPase regulator-interacting protein 1*	*RPGRIP1*	61	US	US	US	US	US
Q63HK3	*Zinc finger protein with KRAB and SCAN domains 2*	*ZKSCAN2*	43	US	US	US	US	US
P25311	*Zinc-alpha-2-glycoprotein*	*AZGP1*	101	US	US	US	US	US
P61769	*Beta-2-microglobulin*	*B2M*	457	1.17	0.16	0.39	0.43	SE
P01034	*Cystatin-C*	*CST3*	444	1.17	0.16	0.12	0.17	SE
P02647	*Apolipoprotein A-I*	*APOA1*	141	1.05	0.05	0.06	0.25	SE
P68871	*Hemoglobin subunit beta*	*HBB*	513	1.04	0.04	0.09	0.34	SE
P02814	*Submaxillary gland androgen-regulated protein 3B*	*SMR3B*	8043	0.96	-0.04	0.03	0.06	SE

Q9UGM3	*Deleted in malignant brain tumors 1 protein*	*DMBT1*	35	0.95	-0.05	0.06	0.24	SE
P06733	*Alpha-enolase*	*ENO1*	262	0.94	-0.06	0.09	0.27	SE
Q96DA0	*Zymogen granule protein 16 homolog B*	*ZG16B*	1084	0.93	-0.07	0.08	0.33	SE
P00738	*Haptoglobin*	*HP*	211	0.90	-0.11	0.08	0.11	SE
P00739	*Haptoglobin-related protein*	*HPR*	23	0.87	-0.14	0.12	0.11	SE
P0DOX7	*Immunoglobulin kappa light chain*	*IGK*	185	0.87	-0.14	0.12	0.11	SE
Q14508	*WAP four-disulfide core domain protein 2*	*WFDC2*	1630	0.86	-0.15	0.74	0.74	SE
Q96DR5	*BPI fold-containing family A member 2*	*BPIFA2*	186	0.84	-0.17	0.12	0.13	SE
P02679	*Fibrinogen gamma chain*	*FGG*	2273	0.84	-0.17	0.1	0.07	SE
P09104	*Gamma-enolase*	*ENO2*	105	0.79	-0.23	0.25	0.2	SE
P13929	*Beta-enolase*	*ENO3*	105	0.79	-0.23	0.16	0.1	SE
P04004	*Vitronectin*	*VTN*	65	0.73	-0.31	0.34	0.19	SE
P69892	*Hemoglobin subunit gamma-2*	*HBG2*	179	0.69	-0.37	0.17	0.05	SE
Q16378	*Proline-rich protein 4*	*PRR4*	903	0.67	-0.4	0.76	0.24	SE
P02100	*Hemoglobin subunit epsilon*	*HBE1*	179	0.67	-0.4	0.12	0.05	SE
P69891	*Hemoglobin subunit gamma-1*	*HBG1*	179	0.66	-0.41	0.13	0.05	SE
P30613	*Pyruvate kinase PKLR*	*PKLR*	29	0.57	-0.57	0.49	0.23	SE

Note: Log(e) ("e" is a constant = 2.71); SD, standard deviation; *p*, statistical significance (adjusted by False Discovery Rate–FDR = 4); ED, Expression differences; ↑ = up-regulated in SS (1-p > 0.95); ↓ = down-regulated in SS (p < 0.05); SE = similar expression in SS and US; bold lines refer to up- or down-regulated proteins by more than 2-fold
