# Supplementary material for: Is There a Difference in the Proteomic Profile of Stimulated and Unstimulated Saliva Samples from Pregnant Women with/without Obesity and Periodontitis?
Source: Cells. 2023 May 14;12(10):1389. doi: 10.3390/cells12101389 (PMC10216649; doi:10.3390/cells12101389)
Supplement: Supplementary file 1 [file cells-12-01389-s001.zip › cells-2289259-supplementary/Supplemetary files/Supplementary file 3.pdf]

Table S3. Expression differences and unique proteins identified in stimulated saliva (SS) and unstimulated saliva (US) of pregnant women with normal BMI but with periodontitis (NP)

Accession number	Protein name	Gene	Score	Fold change	Log(e)	SD	*p*	ED
**P00738**	***Haptoglobin***	***HP***	**114**	**9.68**	**2.27**	**0.06**	**< 0.01**	↑
**P04280**	***Basic salivary proline-rich protein 1***	***PRB1***	**366**	**5.26**	**1.66**	**0.25**	**< 0.01**	↑
**P15516**	***Histatin-3***	***HTN3***	**1825**	**4.90**	**1.59**	**0.4**	**< 0.01**	↑
**P01009**	***Alpha-1-antitrypsin***	***SERPINA1***	**180**	**4.71**	**1.55**	**0.08**	**< 0.01**	↑
**P02812**	***Basic salivary proline-rich protein 2***	***PRB2***	**366**	**4.48**	**1.5**	**0.05**	**< 0.01**	↑
**P02647**	***Apolipoprotein A-I***	***APOA1***	**4432**	**4.10**	**1.41**	**0.05**	**< 0.01**	↑
**P02790**	***Hemopexin***	***HPX***	**153**	**3.25**	**1.18**	**0.05**	**< 0.01**	↑
**P04080**	***Cystatin-B***	***CSTB***	**1239**	**3.16**	**1.15**	**0.08**	**< 0.01**	↑
**Q8N4F0**	***BPI fold-containing family B member 2***	***BPIFB2***	**422**	**2.77**	**1.02**	**0.06**	**< 0.01**	↑
**P02810**	***Salivary acidic proline-rich phosphoprotein 1/2***	***PRH1; PRH2***	**2308**	**2.51**	**0.92**	**0.16**	**< 0.01**	↑
**P69892**	***Hemoglobin subunit gamma-2***	***HBG2***	**271**	**2.39**	**0.87**	**0.13**	**0.01**	↑
**P69891**	***Hemoglobin subunit gamma-1***	***HBG1***	**271**	**2.39**	**0.87**	**0.12**	**< 0.01**	↑
**P02100**	***Hemoglobin subunit epsilon***	***HBE1***	**271**	**2.39**	**0.87**	**0.12**	**< 0.01**	↑
**P02808**	***Statherin***	***STATH***	**2286**	**2.27**	**0.82**	**0.22**	**< 0.01**	↑
**P02679**	***Fibrinogen gamma chain***	***FGG***	**118**	**2.23**	**0.8**	**0.13**	**< 0.01**	↑
**P59666**	***Neutrophil defensin 3***	***DEFA3***	**1062**	**2.12**	**0.75**	**0.06**	**< 0.01**	↑
**P20742**	***Pregnancy zone protein***	***PZP***	**212**	**2.10**	**0.74**	**0.09**	**< 0.01**	↑
**P59665**	***Neutrophil defensin 1***	***DEFA1; DEFA1B***	**1062**	**2.10**	**0.74**	**0.06**	**< 0.01**	↑
**P0DTE8**	***Alpha-amylase 1C***	***AMY1C***	**3994**	**2.08**	**0.73**	**0.01**	**< 0.01**	↑
P01023	*Alpha-2-macroglobulin*	*A2M*	545	1.88	0.63	0.04	< 0.01	↑
P69905	*Hemoglobin subunit alpha*	*HBA1; HBA2*	711	1.88	0.63	0.08	< 0.01	↑
P61769	*Beta-2-microglobulin*	*B2M*	638	1.79	0.58	0.23	0.02	↑
Q9UBC9	*Small proline-rich protein 3*	*SPRR3*	3688	1.67	0.51	0.1	< 0.01	↑
P31025	*Lipocalin-1*	*LCN1*	1539	1.67	0.51	0.05	< 0.01	↑
P0DTE7	*Alpha-amylase 1B*	*AMY1B*	3994	1.67	0.51	0.02	< 0.01	↑
P02675	*Fibrinogen beta chain*	*FGB*	511	1.67	0.51	0.1	< 0.01	↑
P12273	*Prolactin-inducible protein*	*PIP*	1165	1.63	0.49	0.02	< 0.01	↑
P13796	*Plastin-2*	*LCP1*	322	1.58	0.46	0.13	0.04	↑
P07737	*Profilin-1*	*PFN1*	869	1.48	0.39	0.07	< 0.01	↑
P01591	*Immunoglobulin J chain*	*JCHAIN*	697	1.38	0.32	0.06	< 0.01	↑
P02787	*Serotransferrin*	*TF*	118	1.31	0.27	0.04	< 0.01	↑
P05109	*Protein S100-A8*	*S100A8*	452	1.31	0.27	0.04	< 0.01	↑
P04746	*Pancreatic alpha-amylase*	*AMY2A*	2426	1.26	0.23	0.01	< 0.01	↑
P01037	*Cystatin-SN*	*CST1*	3510	1.23	0.21	0.03	< 0.01	↑
P28325	*Cystatin-D*	*CST5*	352	1.20	0.18	0.08	0.03	↑
P09228	*Cystatin-SA*	*CST2*	808	1.13	0.12	0.03	< 0.01	↑
P0DUB6	*Alpha-amylase 1A*	*AMY1A*	3994	0.94	-0.06	0.01	< 0.01	↓
P0DOX5	*Immunoglobulin gamma-1 heavy chain*	*IGG1*	324	0.82	-0.2	0.05	< 0.01	↓
P06733	*Alpha-enolase*	*ENO1*	236	0.82	-0.2	0.07	< 0.01	↓

P19961	*Alpha-amylase 2B*	*AMY2B*	3339	0.82	-0.2	0.01	< 0.01	↓
P01857	*Immunoglobulin heavy constant gamma 1*	*IGHG1*	324	0.82	-0.2	0.05	< 0.01	↓
P01833	*Polymeric immunoglobulin receptor*	*PIGR*	810	0.73	-0.32	0.03	< 0.01	↓
P02814	*Submaxillary gland androgen-regulated protein 3B*	*SMR3B*	1091	0.72	-0.33	0.02	< 0.01	↓
P01871	*Immunoglobulin heavy constant mu*	*IGHM*	288	0.72	-0.33	0.1	0.01	↓
P0DOX6	*Immunoglobulin mu heavy chain*	*IGM*	254	0.64	-0.44	0.1	< 0.01	↓
P01876	*Immunoglobulin heavy constant alpha 1*	*IGHA1*	1300	0.63	-0.46	0.02	< 0.01	↓
P0DOX2	*Immunoglobulin alpha-2 heavy chain*	*IGA2*	449	0.63	-0.47	0.02	< 0.01	↓
P23280	*Carbonic anhydrase 6*	*CA6*	1067	0.63	-0.47	0.09	< 0.01	↓
P01877	*Immunoglobulin heavy constant alpha 2*	*IGHA2*	496	0.63	-0.47	0.01	< 0.01	↓
P01861	*Immunoglobulin heavy constant gamma 4*	*IGHG4*	85	0.59	-0.53	0.1	< 0.01	↓
Q96DA0	*Zymogen granule protein 16 homolog B*	*ZG16B*	1097	0.58	-0.55	0.02	< 0.01	↓
P01036	*Cystatin-S*	*CST4*	6686	0.58	-0.55	0.02	< 0.01	↓
P02768	*Albumin*	*ALB*	2047	0.54	-0.61	0.01	< 0.01	↓
Q96DR5	*BPI fold-containing family A member 2*	*BPIFA2*	221	0.53	-0.64	0.05	< 0.01	↓
P01860	*Immunoglobulin heavy constant gamma 3*	*IGHG3*	85	0.51	-0.68	0.08	< 0.01	↓
**P22079**	***Lactoperoxidase***	***LPO***	**82**	**0.49**	**-0.72**	**0.11**	**< 0.01**	↓
**P0CG38**	***POTE ankyrin domain family member I***	***POTEI***	**257**	**0.47**	**-0.75**	**0.07**	**< 0.01**	↓
**P02788**	***Lactotransferrin***	***LTF***	**58**	**0.47**	**-0.75**	**0.26**	**0.01**	↓
**P0CG39**	***POTE ankyrin domain family member J***	***POTEJ***	**176**	**0.47**	**-0.76**	**0.1**	**< 0.01**	↓
**P68133**	***Actin, alpha skeletal muscle***	***ACTA1***	**904**	**0.45**	**-0.8**	**0.07**	**< 0.01**	↓
**Q8NHQ9**	***ATP-dependent RNA helicase DDX55***	***DDX55***	**169**	**0.44**	**-0.82**	**0.26**	**0.02**	↓
**P63267**	***Actin, gamma-enteric smooth muscle***	***ACTG2***	**904**	**0.43**	**-0.85**	**0.07**	**< 0.01**	↓
**Q5VSP4**	***Putative lipocalin 1-like protein 1***	***LCN1P1***	**967**	**0.43**	**-0.85**	**0.08**	**< 0.01**	↓
**Q6S8J3**	***POTE ankyrin domain family member E***	***POTEE***	**366**	**0.39**	**-0.94**	**0.05**	**< 0.01**	↓
**A5A3E0**	***POTE ankyrin domain family member F***	***POTEF***	**366**	**0.39**	**-0.95**	**0.06**	**< 0.01**	↓
**P68032**	***Actin, alpha cardiac muscle 1***	***ACTC1***	**904**	**0.37**	**-1**	**0.04**	**< 0.01**	↓
**Q9BYX7**	***Putative beta-actin-like protein 3***	***POTEKP***	**109**	**0.36**	**-1.02**	**0.05**	**< 0.01**	↓
**P62736**	***Actin, aortic smooth muscle***	***ACTA2***	**904**	**0.35**	**-1.05**	**0.05**	**< 0.01**	↓
**P63261**	***Actin, cytoplasmic 2***	***ACTG1***	**1384**	**0.34**	**-1.09**	**0.02**	**< 0.01**	↓
**P60709**	***Actin, cytoplasmic 1***	***ACTB***	**1384**	**0.33**	**-1.1**	**0.02**	**< 0.01**	↓
**P02042**	***Hemoglobin subunit delta***	***HBD***	**499**	**0.32**	**-1.13**	**0.05**	**< 0.01**	↓
**A0M8Q6**	***Immunoglobulin lambda constant 7***	***IGLC7***	**320**	**0.23**	**-1.48**	**0.08**	**< 0.01**	↓
**Q8TAX7**	***Mucin-7***	***MUC7***	**842**	**0.20**	**-1.63**	**0.02**	**< 0.01**	↓
**Q16378**	***Proline-rich protein 4***	***PRR4***	**984**	**0.18**	**-1.69**	**0.08**	**0.01**	↓
**Q562R1**	***Beta-actin-like protein 2***	***ACTBL2***	**362**	**0.17**	**-1.75**	**0.02**	**< 0.01**	↓
**P01859**	***Immunoglobulin heavy constant gamma 2***	***IGHG2***	**85**	**0.17**	**-1.8**	**0.06**	**< 0.01**	↓
**P06702**	***Protein S100-A9***	***S100A9***	**344**	**0.13**	**-2.02**	**0.04**	**< 0.01**	↓
**P61626**	***Lysozyme C***	***LYZ***	**3223**	**0.06**	**-2.77**	**0.03**	**< 0.01**	↓
Q9P218	*Collagen alpha-1(XX) chain*	*COL20A1*	30	SS	SS	SS	SS	SS
Q9BWT3	*Poly(A) polymerase gamma*	*PAPOLG*	32	SS	SS	SS	SS	SS
P52209	*6-phosphogluconate dehydrogenase, decarboxylating*	*PGD*	73	US	US	US	US	US
P07108	*Acyl-CoA-binding protein*	*DBI*	195	US	US	US	US	US
Q01518	*Adenylyl cyclase-associated protein 1*	*CAP1*	130	US	US	US	US	US
A8K2U0	*Alpha-2-macroglobulin-like protein 1*	*A2ML1*	38	US	US	US	US	US

P27216	*Annexin A13*	*ANXA13*	392	US	US	US	US	US
P03973	*Antileukoproteinase*	*SLPI*	1387	US	US	US	US	US
P0DP23	*Calmodulin-1*	*CALM1*	252	US	US	US	US	US
P0DP24	*Calmodulin-2*	*CALM2*	252	US	US	US	US	US
P0DP25	*Calmodulin-3*	*CALM3*	252	US	US	US	US	US
Q8NEL0	*Coiled-coil domain-containing protein 54*	*CCDC54*	185	US	US	US	US	US
P54108	*Cysteine-rich secretory protein 3*	*CRISP3*	74	US	US	US	US	US
Q99543	*DnaJ homolog subfamily C member 2*	*DNAJC2*	51	US	US	US	US	US
P11021	*Endoplasmic reticulum chaperone BiP*	*HSPA5*	30	US	US	US	US	US
P15311	*Ezrin*	*EZR*	20	US	US	US	US	US
Q9P2Q2	*FERM domain-containing protein 4A*	*FRMD4A*	24	US	US	US	US	US
P04075	*Fructose-bisphosphate aldolase A*	*ALDOA*	194	US	US	US	US	US
Q5RHP9	*Glutamate-rich protein 3*	*ERICH3*	41	US	US	US	US	US
Q9UJ14	*Glutathione hydrolase 7*	*GGT7*	64	US	US	US	US	US
P09211	*Glutathione S-transferase P*	*GSTP1*	247	US	US	US	US	US
O14556	*Glyceraldehyde-3-phosphate dehydrogenase, testis-specific*	*GAPDHS*	93	US	US	US	US	US
Q8NBJ4	*Golgi membrane protein 1*	*GOLM1*	63	US	US	US	US	US
P34931	*Heat shock 70 kDa protein 1-like*	*HSPA1L*	181	US	US	US	US	US
P0DMV8	*Heat shock 70 kDa protein 1A*	*HSPA1A*	196	US	US	US	US	US
P0DMV9	*Heat shock 70 kDa protein 1B*	*HSPA1B*	184	US	US	US	US	US
P17066	*Heat shock 70 kDa protein 6*	*HSPA6*	52	US	US	US	US	US
P11142	*Heat shock cognate 71 kDa protein*	*HSPA8*	67	US	US	US	US	US
P54652	*Heat shock-related 70 kDa protein 2*	*HSPA2*	58	US	US	US	US	US
P02008	*Hemoglobin subunit zeta*	*HBZ*	710	US	US	US	US	US
P01764	*Immunoglobulin heavy variable 3-23*	*IGHV3-23*	928	US	US	US	US	US
P01768	*Immunoglobulin heavy variable 3-30*	*IGHV3-30*	928	US	US	US	US	US
P0DP02	*Immunoglobulin heavy variable 3-30-3*	*IGHV3-30-3*	928	US	US	US	US	US
P0DP03	*Immunoglobulin heavy variable 3-30-5*	*IGHV3-30-5*	928	US	US	US	US	US
P01772	*Immunoglobulin heavy variable 3-33*	*IGHV3-33*	928	US	US	US	US	US
P01767	*Immunoglobulin heavy variable 3-53*	*IGHV3-53*	928	US	US	US	US	US
A0A0C4DH42	*Immunoglobulin heavy variable 3-66*	*IGHV3-66*	928	US	US	US	US	US
A0A0B4J1X5	*Immunoglobulin heavy variable 3-74*	*IGHV3-74*	928	US	US	US	US	US
A0A075B6P5	*Immunoglobulin kappa variable 2-28*	*IGKV2-28*	146	US	US	US	US	US
A2NJV5	*Immunoglobulin kappa variable 2-29*	*IGKV2-29*	146	US	US	US	US	US
P06310	*Immunoglobulin kappa variable 2-30*	*IGKV2-30*	146	US	US	US	US	US
A0A087WW87	*Immunoglobulin kappa variable 2-40*	*IGKV2-40*	146	US	US	US	US	US
A0A0A0MRZ7	*Immunoglobulin kappa variable 2D-26*	*IGKV2D-26*	146	US	US	US	US	US
P01615	*Immunoglobulin kappa variable 2D-28*	*IGKV2D-28*	160	US	US	US	US	US
A0A075B6S2	*Immunoglobulin kappa variable 2D-29*	*IGKV2D-29*	146	US	US	US	US	US
A0A075B6S6	*Immunoglobulin kappa variable 2D-30*	*IGKV2D-30*	146	US	US	US	US	US
P01614	*Immunoglobulin kappa variable 2D-40*	*IGKV2D-40*	146	US	US	US	US	US
P04433	*Immunoglobulin kappa variable 3-11*	*IGKV3-11*	578	US	US	US	US	US
A0A0A0MRZ8	*Immunoglobulin kappa variable 3D-11*	*IGKV3D-11*	578	US	US	US	US	US
P00338	*L-lactate dehydrogenase A chain*	*LDHA*	117	US	US	US	US	US
Q6ZMR3	*L-lactate dehydrogenase A-like 6A*	*LDHAL6A*	56	US	US	US	US	US

P07195	*L-lactate dehydrogenase B chain*	*LDHB*	56	US	US	US	US	US
P07864	*L-lactate dehydrogenase C chain*	*LDHC*	56	US	US	US	US	US
Q6B0I6	*Lysine-specific demethylase 4D*	*KDM4D*	104	US	US	US	US	US
P14780	*Matrix metalloproteinase-9*	*MMP9*	82	US	US	US	US	US
P26038	*Moesin*	*MSN*	50	US	US	US	US	US
Q02817	*Mucin-2*	*MUC2*	20	US	US	US	US	US
P80188	*Neutrophil gelatinase-associated lipocalin*	*LCN2*	366	US	US	US	US	US
P47874	*Olfactory marker protein*	*OMP*	102	US	US	US	US	US
P62937	*Peptidyl-prolyl cis-trans isomerase A*	*PPIA*	357	US	US	US	US	US
Q14651	*Plastin-1*	*PLS1*	33	US	US	US	US	US
P13797	*Plastin-3*	*PLS3*	86	US	US	US	US	US
Q9Y2S7	*Polymerase delta-interacting protein 2*	*POLDIP2*	70	US	US	US	US	US
Q9UQ80	*Proliferation-associated protein 2G4*	*PA2G4*	41	US	US	US	US	US
Q6MZM9	*Proline-rich protein 27*	*PRR27*	248	US	US	US	US	US
Q6P5S2	*Protein LEG1 homolog*	*LEG1*	404	US	US	US	US	US
P80511	*Protein S100-A12*	*S100A12*	209	US	US	US	US	US
P48741	*Putative heat shock 70 kDa protein 7*	*HSPA7*	52	US	US	US	US	US
P35241	*Radixin*	*RDX*	19	US	US	US	US	US
Q9H299	*SH3 domain-binding glutamic acid-rich-like protein 3*	*SH3BGRL3*	354	US	US	US	US	US
P35326	*Small proline-rich protein 2A*	*SPRR2A*	526	US	US	US	US	US
P35325	*Small proline-rich protein 2B*	*SPRR2B*	245	US	US	US	US	US
P22532	*Small proline-rich protein 2D*	*SPRR2D*	245	US	US	US	US	US
P22531	*Small proline-rich protein 2E*	*SPRR2E*	245	US	US	US	US	US
Q9BYE4	*Small proline-rich protein 2G*	*SPRR2G*	245	US	US	US	US	US
Q6UWP8	*Suprabasin*	*SBSN*	60	US	US	US	US	US
P10599	*Thioredoxin*	*TXN*	143	US	US	US	US	US
P26639	*Threonine--tRNA ligase 1, cytoplasmic*	*TARS1*	92	US	US	US	US	US
Q9Y4F4	*TOG array regulator of axonemal microtubules protein 1*	*TOGARAM1*	97	US	US	US	US	US
P37837	*Transaldolase*	*TALDO1*	152	US	US	US	US	US
P51809	*Vesicle-associated membrane protein 7*	*VAMP7*	100	US	US	US	US	US
Q14508	*WAP four-disulfide core domain protein 2*	*WFDC2*	108	US	US	US	US	US
P02774	*Vitamin D-binding protein*	*GC*	137	1.93	0.66	0.4	0.08	SE
P01034	*Cystatin-C*	*CST3*	141	1.15	0.14	0.11	0.15	SE
P01834	*Immunoglobulin kappa constant*	*IGKC*	3138	1.13	0.12	0.07	0.07	SE
P04406	*Glyceraldehyde-3-phosphate dehydrogenase*	*GAPDH*	1223	1.04	0.04	0.13	0.37	SE
P30613	*Pyruvate kinase PKLR*	*PKLR*	129	1.01	0.01	0.2	0.5	SE
P29401	*Transketolase*	*TKT*	171	0.96	-0.04	0.12	0.4	SE
Q9UBG3	*Cornulin*	*CRNN*	258	0.96	-0.04	0.12	0.42	SE
P24158	*Myeloblastin*	*PRTN3*	68	0.88	-0.13	0.19	0.25	SE
P0DOX7	*Immunoglobulin kappa light chain*	*IGK*	2520	0.86	-0.15	0.44	0.54	SE
P15515	*Histatin-1*	*HTN1*	202	0.79	-0.23	0.39	0.53	SE
P09104	*Gamma-enolase*	*ENO2*	32	0.76	-0.27	0.24	0.18	SE
P13929	*Beta-enolase*	*ENO3*	50	0.75	-0.29	0.27	0.14	SE
Q8TDL5	*BPI fold-containing family B member 1*	*BPIFB1*	86	0.71	-0.34	0.21	0.09	SE

Note: Log(e) ("e" is a constant = 2.71); SD, standard deviation; *p*, statistical significance (adjusted by False Discovery Rate–FDR = 4); ED, Expression differences; ↑ = up-regulated in SS ($1-p > 0.95$); ↓ = down-regulated in SS ($p < 0.05$); SE = similar expression in SS and US; bold lines refer to up- or down-regulated proteins by more than 2-fold
