# Supplementary material for: Is There a Difference in the Proteomic Profile of Stimulated and Unstimulated Saliva Samples from Pregnant Women with/without Obesity and Periodontitis?
Source: Cells. 2023 May 14;12(10):1389. doi: 10.3390/cells12101389 (PMC10216649; doi:10.3390/cells12101389)
Supplement: Supplementary file 1 [file cells-12-01389-s001.zip › cells-2289259-supplementary/Supplemetary files/Supplementary file 4.pdf]

Table S4. Expression differences and unique proteins identified in stimulated saliva (SS) and unstimulated saliva (US) of pregnant women with normal BMI and without periodontitis (NWP)

Accession number	Protein name	Gene	Score	Fold change	Log(e)	SD	*p*	ED
**P02814**	***Submaxillary gland androgen-regulated protein 3B***	***SMR3B***	**51408**	**2.94**	**1.08**	**0.02**	**< 0.01**	↑
**P61769**	***Beta-2-microglobulin***	***B2M***	**132**	**2.94**	**1.08**	**0.1**	**< 0.01**	↑
**P06702**	***Protein S100-A9***	***S100A9***	**181**	**2.53**	**0.93**	**0.08**	**< 0.01**	↑
**Q8TAX7**	***Mucin-7***	***MUC7***	**1497**	**2.27**	**0.82**	**0.04**	**< 0.01**	↑
**P0DOX8**	***Immunoglobulin lambda-1 light chain***	***IGL1***	**3256**	**2.08**	**0.73**	**0.06**	**< 0.01**	↑
P68871	*Hemoglobin subunit beta*	*HBB*	151	1.80	0.59	0.3	< 0.01	↑
P28325	*Cystatin-D*	*CST5*	333	1.67	0.51	0.05	< 0.01	↑
P04746	*Pancreatic alpha-amylase*	*AMY2A*	28592	1.63	0.49	0.01	< 0.01	↑
Q9UGM3	*Deleted in malignant brain tumors 1 protein*	*DMBT1*	393	1.45	0.37	0.08	< 0.01	↑
P01871	*Immunoglobulin heavy constant mu*	*IGHM*	118	1.39	0.33	0.12	< 0.01	↑
Q9UBC9	*Small proline-rich protein 3*	*SPRR3*	1968	1.35	0.3	0.15	0.04	↑
P0DOX6	*Immunoglobulin mu heavy chain*	*IGM*	118	1.31	0.27	0.13	0.04	↑
P19961	*Alpha-amylase 2B*	*AMY2B*	35774	1.30	0.26	0.01	< 0.01	↑
P0DUB6	*Alpha-amylase 1A*	*AMY1A*	41555	1.27	0.24	0.01	< 0.01	↑
P0DTE8	*Alpha-amylase 1C*	*AMY1C*	41555	1.22	0.2	0.01	< 0.01	↑
P0DTE7	*Alpha-amylase 1B*	*AMY1B*	41555	1.20	0.18	0.02	< 0.01	↑
P0DOX7	*Immunoglobulin kappa light chain*	*IGK*	276	1.19	0.17	0.07	0.01	↑
P01834	*Immunoglobulin kappa constant*	*IGKC*	1907	1.09	0.09	0.04	0.01	↑
P01037	*Cystatin-SN*	*CST1*	10770	0.86	-0.15	0.03	< 0.01	↓
Q96DR5	*BPI fold-containing family A member 2*	*BPIFA2*	944	0.82	-0.2	0.06	< 0.01	↓
P0CG04	*Immunoglobulin lambda constant 1*	*IGLC1*	3086	0.76	-0.27	0.07	< 0.01	↓
P02788	*Lactotransferrin*	*LTF*	425	0.75	-0.29	0.16	0.04	↓
P0CF74	*Immunoglobulin lambda constant 6*	*IGLC6*	3630	0.75	-0.29	0.08	< 0.01	↓
P0DOY2	*Immunoglobulin lambda constant 2*	*IGLC2*	4884	0.74	-0.3	0.1	< 0.01	↓
P09228	*Cystatin-SA*	*CST2*	3237	0.72	-0.33	0.02	< 0.01	↓
P01860	*Immunoglobulin heavy constant gamma 3*	*IGHG3*	103	0.66	-0.42	0.17	0.01	↓
P60709	*Actin, cytoplasmic 1*	*ACTB*	1017	0.65	-0.43	0.04	< 0.01	↓
P63261	*Actin, cytoplasmic 2*	*ACTG1*	1014	0.64	-0.44	0.05	< 0.01	↓
P01833	*Polymeric immunoglobulin receptor*	*PIGR*	16349	0.64	-0.44	0.03	< 0.01	↓
P01034	*Cystatin-C*	*CST3*	3385	0.64	-0.44	0.07	< 0.01	↓
P63267	*Actin, gamma-enteric smooth muscle*	*ACTG2*	884	0.64	-0.45	0.05	< 0.01	↓
P0DOX5	*Immunoglobulin gamma-1 heavy chain*	*IGG1*	184	0.63	-0.47	0.09	< 0.01	↓
P68133	*Actin, alpha skeletal muscle*	*ACTA1*	884	0.62	-0.48	0.06	< 0.01	↓
P68032	*Actin, alpha cardiac muscle 1*	*ACTC1*	884	0.62	-0.48	0.07	< 0.01	↓
P62736	*Actin, aortic smooth muscle*	*ACTA2*	884	0.62	-0.48	0.05	< 0.01	↓
Q8N4F0	*BPI fold-containing family B member 2*	*BPIFB2*	612	0.61	-0.5	0.15	0.01	↓
B9A064	*Immunoglobulin lambda-like polypeptide 5*	*IGLL5*	3303	0.60	-0.51	0.22	0.03	↓
Q16378	*Proline-rich protein 4*	*PRR4*	3936	0.60	-0.51	0.07	< 0.01	↓
P01877	*Immunoglobulin heavy constant alpha 2*	*IGHA2*	4178	0.59	-0.52	0.02	< 0.01	↓
P0DOX2	*Immunoglobulin alpha-2 heavy chain*	*IGA2*	4092	0.59	-0.53	0.03	< 0.01	↓

P05109	*Protein S100-A8*	*S100A8*	250	0.59	-0.53	0.1	< 0.01	↓
P01876	*Immunoglobulin heavy constant alpha 1*	*IGHA1*	9432	0.58	-0.54	0.02	< 0.01	↓
P01857	*Immunoglobulin heavy constant gamma 1*	*IGHG1*	184	0.58	-0.54	0.08	< 0.01	↓
P02787	*Serotransferrin*	*TF*	391	0.58	-0.55	0.08	< 0.01	↓
P0DOY3	*Immunoglobulin lambda constant 3*	*IGLC3*	4884	0.55	-0.6	0.16	< 0.01	↓
P02768	*Albumin*	*ALB*	6357	0.54	-0.62	0.02	< 0.01	↓
P12273	*Prolactin-inducible protein*	*PIP*	25257	0.54	-0.62	0.03	< 0.01	↓
P22079	*Lactoperoxidase*	*LPO*	617	0.53	-0.64	0.12	< 0.01	↓
**Q9BYX7**	***Putative beta-actin-like protein 3***	***POTEKP***	**254**	**0.50**	**-0.7**	**0.09**	**< 0.01**	↓
**P01036**	***Cystatin-S***	***CST4***	**16480**	**0.50**	**-0.7**	**0.02**	**< 0.01**	↓
**A0M8Q6**	***Immunoglobulin lambda constant 7***	***IGLC7***	**2152**	**0.48**	**-0.74**	**0.23**	**< 0.01**	↓
**P07737**	***Profilin-1***	***PFN1***	**859**	**0.46**	**-0.77**	**0.16**	**< 0.01**	↓
**P61626**	***Lysozyme C***	***LYZ***	**3743**	**0.45**	**-0.8**	**0.16**	**< 0.01**	↓
**P02810**	***Salivary acidic proline-rich phosphoprotein 1/2***	***PRH1; PRH2***	**3931**	**0.43**	**-0.85**	**0.02**	**< 0.01**	↓
**Q562R1**	***Beta-actin-like protein 2***	***ACTBL2***	**626**	**0.43**	**-0.85**	**0.09**	**< 0.01**	↓
**A5A3E0**	***POTE ankyrin domain family member F***	***POTEF***	**337**	**0.42**	**-0.86**	**0.07**	**< 0.01**	↓
**P37837**	***Transaldolase***	***TALDO1***	**199**	**0.42**	**-0.87**	**0.23**	**< 0.01**	↓
**Q96DA0**	***Zymogen granule protein 16 homolog B***	***ZG16B***	**22215**	**0.42**	**-0.87**	**0.03**	**< 0.01**	↓
**Q6S8J3**	***POTE ankyrin domain family member E***	***POTEE***	**337**	**0.41**	**-0.88**	**0.05**	**< 0.01**	↓
**P0CG38**	***POTE ankyrin domain family member I***	***POTEI***	**83**	**0.36**	**-1.02**	**0.09**	**< 0.01**	↓
**P01861**	***Immunoglobulin heavy constant gamma 4***	***IGHG4***	**122**	**0.32**	**-1.13**	**0.29**	**< 0.01**	↓
**P01859**	***Immunoglobulin heavy constant gamma 2***	***IGHG2***	**30**	**0.31**	**-1.17**	**0.29**	**< 0.01**	↓
**P0CG39**	***POTE ankyrin domain family member J***	***POTEJ***	**83**	**0.30**	**-1.22**	**0.17**	**< 0.01**	↓
**P23280**	***Carbonic anhydrase 6***	***CA6***	**1177**	**0.25**	**-1.38**	**0.03**	**< 0.01**	↓
**P31025**	***Lipocalin-1***	***LCN1***	**5807**	**0.10**	**-2.3**	**0.03**	**< 0.01**	↓
**Q5VSP4**	***Putative lipocalin 1-like protein 1***	***LCN1P1***	**4592**	**0.10**	**-2.33**	**0.03**	**< 0.01**	↓
Q9H3E2	*Sorting nexin-25*	*SNX25*	50	SS	SS	SS	SS	SS
Q8TAF7	*Zinc finger protein 461*	*ZNF461*	61	SS	SS	SS	SS	SS
Q15118	*[Pyruvate dehydrogenase (acetyl-transferring)] kinase isozyme 1, mitochondrial*	*PDK1*	72	US	US	US	US	US
P52209	*6-phosphogluconate dehydrogenase, decarboxylating*	*PGD*	144	US	US	US	US	US
P07108	*Acyl-CoA-binding protein*	*DBI*	297	US	US	US	US	US
Q01518	*Adenylyl cyclase-associated protein 1*	*CAP1*	135	US	US	US	US	US
P01011	*Alpha-1-antichymotrypsin*	*SERPINA3*	68	US	US	US	US	US
P01009	*Alpha-1-antitrypsin*	*SERPINA1*	95	US	US	US	US	US
P01023	*Alpha-2-macroglobulin*	*A2M*	27	US	US	US	US	US
A8K2U0	*Alpha-2-macroglobulin-like protein 1*	*A2ML1*	109	US	US	US	US	US
P04920	*Anion exchange protein 2*	*SLC4A2*	45	US	US	US	US	US
P03973	*Antileukoproteinase*	*SLPI*	1419	US	US	US	US	US
P02647	*Apolipoprotein A-I*	*APOA1*	171	US	US	US	US	US
Q9H115	*Beta-soluble NSF attachment protein*	*NAPB*	84	US	US	US	US	US
Q8TDL5	*BPI fold-containing family B member 1*	*BPIFB1*	912	US	US	US	US	US
P27482	*Calmodulin-like protein 3*	*CALML3*	417	US	US	US	US	US
P23528	*Cofilin-1*	*CFL1*	269	US	US	US	US	US

P01024	*Complement C3*	*C3*	45	US	US	US	US	US
P54108	*Cysteine-rich secretory protein 3*	*CRISP3*	885	US	US	US	US	US
Q02487	*Desmocollin-2*	*DSC2*	38	US	US	US	US	US
Q92616	*eIF-2-alpha kinase activator GCN1*	*GCN1*	57	US	US	US	US	US
P11021	*Endoplasmic reticulum chaperone BiP*	*HSPA5*	101	US	US	US	US	US
Q9GZZ8	*Extracellular glycoprotein lacritin*	*LACRT*	2060	US	US	US	US	US
Q01469	*Fatty acid-binding protein 5*	*FABP5*	557	US	US	US	US	US
Q5W0V3	*FHF complex subunit HOOK interacting protein 2A*	*FHIP2A*	159	US	US	US	US	US
P04075	*Fructose-bisphosphate aldolase A*	*ALDOA*	182	US	US	US	US	US
Q08380	*Galectin-3-binding protein*	*LGALS3BP*	92	US	US	US	US	US
P06396	*Gelsolin*	*GSN*	47	US	US	US	US	US
Q9UJ14	*Glutathione hydrolase 7*	*GGT7*	123	US	US	US	US	US
P09211	*Glutathione S-transferase P*	*GSTP1*	1006	US	US	US	US	US
P04406	*Glyceraldehyde-3-phosphate dehydrogenase*	*GAPDH*	705	US	US	US	US	US
P00738	*Haptoglobin*	*HP*	132	US	US	US	US	US
P00739	*Haptoglobin-related protein*	*HPR*	43	US	US	US	US	US
P34931	*Heat shock 70 kDa protein 1-like*	*HSPA1L*	90	US	US	US	US	US
P0DMV8	*Heat shock 70 kDa protein 1A*	*HSPA1A*	116	US	US	US	US	US
P0DMV9	*Heat shock 70 kDa protein 1B*	*HSPA1B*	116	US	US	US	US	US
P17066	*Heat shock 70 kDa protein 6*	*HSPA6*	95	US	US	US	US	US
P11142	*Heat shock cognate 71 kDa protein*	*HSPA8*	82	US	US	US	US	US
P54652	*Heat shock-related 70 kDa protein 2*	*HSPA2*	101	US	US	US	US	US
P02790	*Hemopexin*	*HPX*	108	US	US	US	US	US
Q9Y6R7	*IgGFc-binding protein*	*FCGBP*	45	US	US	US	US	US
A0A075B6P5	*Immunoglobulin kappa variable 2-28*	*IGKV2-28*	236	US	US	US	US	US
A2NJV5	*Immunoglobulin kappa variable 2-29*	*IGKV2-29*	236	US	US	US	US	US
P06310	*Immunoglobulin kappa variable 2-30*	*IGKV2-30*	236	US	US	US	US	US
A0A087WW87	*Immunoglobulin kappa variable 2-40*	*IGKV2-40*	236	US	US	US	US	US
A0A0A0MRZ7	*Immunoglobulin kappa variable 2D-26*	*IGKV2D-26*	236	US	US	US	US	US
P01615	*Immunoglobulin kappa variable 2D-28*	*IGKV2D-28*	236	US	US	US	US	US
A0A075B6S2	*Immunoglobulin kappa variable 2D-29*	*IGKV2D-29*	236	US	US	US	US	US
A0A075B6S6	*Immunoglobulin kappa variable 2D-30*	*IGKV2D-30*	236	US	US	US	US	US
P01614	*Immunoglobulin kappa variable 2D-40*	*IGKV2D-40*	236	US	US	US	US	US
P18510	*Interleukin-1 receptor antagonist protein*	*IL1RN*	113	US	US	US	US	US
O95274	*Ly6/PLAUR domain-containing protein 3*	*LYPD3*	118	US	US	US	US	US
Q96DR8	*Mucin-like protein 1*	*MUCL1*	606	US	US	US	US	US
P24158	*Myeloblastin*	*PRTN3*	177	US	US	US	US	US
Q15406	*Nuclear receptor subfamily 6 group A member 1*	*NR6A1*	48	US	US	US	US	US
P62937	*Peptidyl-prolyl cis-trans isomerase A*	*PPIA*	226	US	US	US	US	US
Q9Y536	*Peptidyl-prolyl cis-trans isomerase A-like 4A*	*PPIAL4A*	54	US	US	US	US	US
P00558	*Phosphoglycerate kinase 1*	*PGK1*	83	US	US	US	US	US
P07205	*Phosphoglycerate kinase 2*	*PGK2*	83	US	US	US	US	US
P13796	*Plastin-2*	*LCP1*	123	US	US	US	US	US
P07602	*Prosaposin*	*PSAP*	116	US	US	US	US	US
Q6P5S2	*Protein LEG1 homolog*	*LEG1*	2046	US	US	US	US	US

A8MUU1	*Putative fatty acid-binding protein 5-like protein 3*	*FABP5P3*	79	US	US	US	US	US
P48741	*Putative heat shock 70 kDa protein 7*	*HSPA7*	95	US	US	US	US	US
P30613	*Pyruvate kinase PKLR*	*PKLR*	91	US	US	US	US	US
P14618	*Pyruvate kinase PKM*	*PKM*	135	US	US	US	US	US
P52566	*Rho GDP-dissociation inhibitor 2*	*ARHGDIB*	370	US	US	US	US	US
Q53EL9	*Seizure protein 6 homolog*	*SEZ6*	70	US	US	US	US	US
P29508	*Serpin B3*	*SERPINB3*	82	US	US	US	US	US
P48594	*Serpin B4*	*SERPINB4*	82	US	US	US	US	US
Q9H299	*SH3 domain-binding glutamic acid-rich-like protein 3*	*SH3BGRL3*	305	US	US	US	US	US
P35326	*Small proline-rich protein 2A*	*SPRR2A*	733	US	US	US	US	US
P35325	*Small proline-rich protein 2B*	*SPRR2B*	373	US	US	US	US	US
P22532	*Small proline-rich protein 2D*	*SPRR2D*	373	US	US	US	US	US
P22531	*Small proline-rich protein 2E*	*SPRR2E*	373	US	US	US	US	US
Q96RM1	*Small proline-rich protein 2F*	*SPRR2F*	75	US	US	US	US	US
Q9BYE4	*Small proline-rich protein 2G*	*SPRR2G*	347	US	US	US	US	US
P10599	*Thioredoxin*	*TXN*	4451	US	US	US	US	US
P20061	*Transcobalamin-1*	*TCN1*	201	US	US	US	US	US
P29401	*Transketolase*	*TKT*	89	US	US	US	US	US
P60174	*Triosephosphate isomerase*	*TPI1*	181	US	US	US	US	US
Q8IXR9	*Uncharacterized protein C12orf56*	*C12orf56*	54	US	US	US	US	US
P11684	*Uteroglobin*	*SCGB1A1*	3363	US	US	US	US	US
Q14508	*WAP four-disulfide core domain protein 2*	*WFDC2*	2229	US	US	US	US	US
P25311	*Zinc-alpha-2-glycoprotein*	*AZGP1*	290	US	US	US	US	US
P15516	*Histatin-3*	*HTN3*	958	3.94	1.37	0.45	0.06	SE
P02100	*Hemoglobin subunit epsilon*	*HBE1*	119	2.03	0.71	0.69	0.36	SE
P69891	*Hemoglobin subunit gamma-1*	*HBG1*	119	1.90	0.64	0.65	0.4	SE
P69892	*Hemoglobin subunit gamma-2*	*HBG2*	119	1.80	0.59	0.65	0.44	SE
P04080	*Cystatin-B*	*CSTB*	5550	1.34	0.29	0.19	0.12	SE
Q8WXA9	*Splicing regulatory glutamine/lysine-rich protein 1*	*SREK1*	63	1.19	0.17	0.21	0.29	SE
P02042	*Hemoglobin subunit delta*	*HBD*	151	1.14	0.13	0.14	0.21	SE
P01591	*Immunoglobulin J chain*	*JCHAIN*	9177	0.91	-0.09	0.08	0.12	SE
P59666	*Neutrophil defensin 3*	*DEFA3*	112	0.79	-0.24	0.18	0.12	SE
P09104	*Gamma-enolase*	*ENO2*	124	0.78	-0.25	0.3	0.21	SE
P69905	*Hemoglobin subunit alpha*	*HBA1; HBA2*	95	0.78	-0.25	0.14	0.06	SE
P59665	*Neutrophil defensin 1*	*DEFA1; DEFA1B*	112	0.77	-0.26	0.17	0.12	SE
P06744	*Glucose-6-phosphate isomerase*	*GPI*	96	0.68	-0.39	0.45	0.23	SE
P15515	*Histatin-1*	*HTN1*	17313	0.64	-0.45	0.7	0.3	SE
P02808	*Statherin*	*STATH*	46710	0.54	-0.62	0.44	0.23	SE

Note: Log(e) ("e" is a constant = 2.71); SD, standard deviation; *p*, statistical significance (adjusted by False Discovery Rate–FDR = 4); ED, Expression differences; ↑ = up-regulated in SS (1-*p* > 0.95); ↓ = down-regulated in SS(*p* < 0.05); SE = similar expression in SS and US; bold lines refer to up- or down-regulated proteins by more than 2-fold
